# The Transcriptome of the Human Pathogen *Trypanosoma brucei* at Single-Nucleotide Resolution

**DOI:** 10.1371/journal.ppat.1001090

**Published:** 2010-09-09

**Authors:** Nikolay G. Kolev, Joseph B. Franklin, Shai Carmi, Huafang Shi, Shulamit Michaeli, Christian Tschudi

**Affiliations:** 1 School of Public Health, Yale University, New Haven, Connecticut, United States of America; 2 Department of Cell Biology, School of Medicine, Yale University, New Haven, Connecticut, United States of America; 3 The Mina and Everard Goodman Faculty of Life Sciences, Bar Ilan University, Ramat-Gan, Israel; 4 Department of Internal Medicine, School of Medicine, Yale University, New Haven, Connecticut, United States of America; Washington University School of Medicine, United States of America

## Abstract

The genome of *Trypanosoma brucei*, the causative agent of African trypanosomiasis, was published five years ago, yet identification of all genes and their transcripts remains to be accomplished. Annotation is challenged by the organization of genes transcribed by RNA polymerase II (Pol II) into long unidirectional gene clusters with no knowledge of how transcription is initiated. Here we report a single-nucleotide resolution genomic map of the *T. brucei* transcriptome, adding 1,114 new transcripts, including 103 non-coding RNAs, confirming and correcting many of the annotated features and revealing an extensive heterogeneity of 5′ and 3′ ends. Some of the new transcripts encode polypeptides that are either conserved in *T. cruzi* and *Leishmania major* or were previously detected in mass spectrometry analyses. High-throughput RNA sequencing (RNA-Seq) was sensitive enough to detect transcripts at putative Pol II transcription initiation sites. Our results, as well as recent data from the literature, indicate that transcription initiation is not solely restricted to regions at the beginning of gene clusters, but may occur at internal sites. We also provide evidence that transcription at all putative initiation sites in *T. brucei* is bidirectional, a recently recognized fundamental property of eukaryotic promoters. Our results have implications for gene expression patterns in other important human pathogens with similar genome organization (*Trypanosoma cruzi*, *Leishmania* sp.) and revealed heterogeneity in pre-mRNA processing that could potentially contribute to the survival and success of the parasite population in the insect vector and the mammalian host.

## Author Summary

Identifying genes essential for survival in the host is fundamental to unraveling the biology of human pathogens and understanding mechanisms of pathogenesis. The protozoan parasite *Trypanosoma brucei* causes devastating diseases in humans and animals in sub-Saharan Africa, and the publication in 2005 of the genome sequence provided the first glance at the coding potential of this organism. Although at present there is a catalogue of predicted protein coding genes, the challenge remains to identify all authentic genes, including their boundaries. We used next generation RNA sequencing (RNA-Seq) to map transcribed regions and RNA polymerase II transcription initiation sites on a genome-wide scale. This approach allowed us to improve and correct the current annotation, to reveal a widespread heterogeneity of RNA processing sites (*trans*-splicing and polyadenylation) and to estimate that most genes are expressed at levels corresponding to 1 to 10 mRNAs per cell. Our data indicate that different transcript forms representing the same gene are present stochastically within the mRNA population. This unanticipated scenario may contribute to determining gene expression landscapes to adapt to different environments in the parasite life cycle.

## Introduction

One of the milestones towards an understanding and possible treatment of human African trypanosomiasis was the publication of the genome sequence of its causative agent, the protozoan parasite *Trypanosoma brucei* [1]. The availability of the potential coding capacity provided a first opportunity to comprehensively annotate the *T. brucei* genome. This initial analysis of the 11 megabase-sized chromosomes predicted 9,068 protein-coding genes, including about 900 pseudogenes. As of April 2010, the catalogue of annotated protein-coding genes has increased to 10,533 (TriTrypDB) [2], yet a major challenge remains to identify all authentic genes, including their boundaries. Such information is central to determining the timing and regulation of gene expression in different developmental stages and the identification of functional elements.

The genomes of *T. brucei* and related trypanosomatids are organized into long unidirectional gene clusters that are transcribed by RNA polymerase II (Pol II) into polycistronic primary transcripts [3]–[6]. However, the sites and mechanism of transcription initiation and termination for protein-coding genes are largely unknown. Individual mRNAs are matured by coupled *trans*-splicing and polyadenylation [7], [8]. In *trans*-splicing, the spliced leader RNA (SL RNA) donates the 39-nt SL sequence that is attached to the 5′ end of all mRNAs and provides the 5′ cap structure for the mRNA [9]. This intermolecular splicing mechanism is an ancient trait in eukaryotes and is found in many protozoa, nematodes, chordates and other organisms [10] and involves molecular mechanisms similar to *cis*-splicing [11]. The sequence signals that determine the *trans*-splice acceptor site appear to consist only of AG dinucleotide at the site for exon junction preceded by a polypyrimidine tract of varying length [8], [11]–[13]. Additional nucleotides downstream of the splice site appear to modulate the efficiency of *trans*-splicing [12], [14], however, only a few cases have been studied. *Trans*-splicing is spatially and temporally coupled to polyadenylation of the upstream mRNA in the polycistron [7], [8], [15]. Poly(A) tail addition has been shown to occur at one of several closely spaced positions with a possible preference for A residues, based on a limited set of cDNAs [13]. Computational approaches have been used to predict *trans*-splice and polyadenylation sites in *T. brucei* [13], [16] and when we started this study, there were no comprehensive studies of *bona fide* pre-mRNA processing sites in trypanosomatids. Thus, the emergence of next-generation sequencing technologies offered a unique opportunity to provide a complete catalogue of 5′ and 3′ end processing sites and to validate the annotated features of the *T. brucei* genome at the transcript level. In the current study, as well as in a recent publication by Siegel et al. [17], high-throughput RNA sequencing (RNA-Seq) was used to generate a *T. brucei* transcriptome map at single-nucleotide resolution.

## Results

### Data sets obtained by RNA-Seq

**Figure 1:**
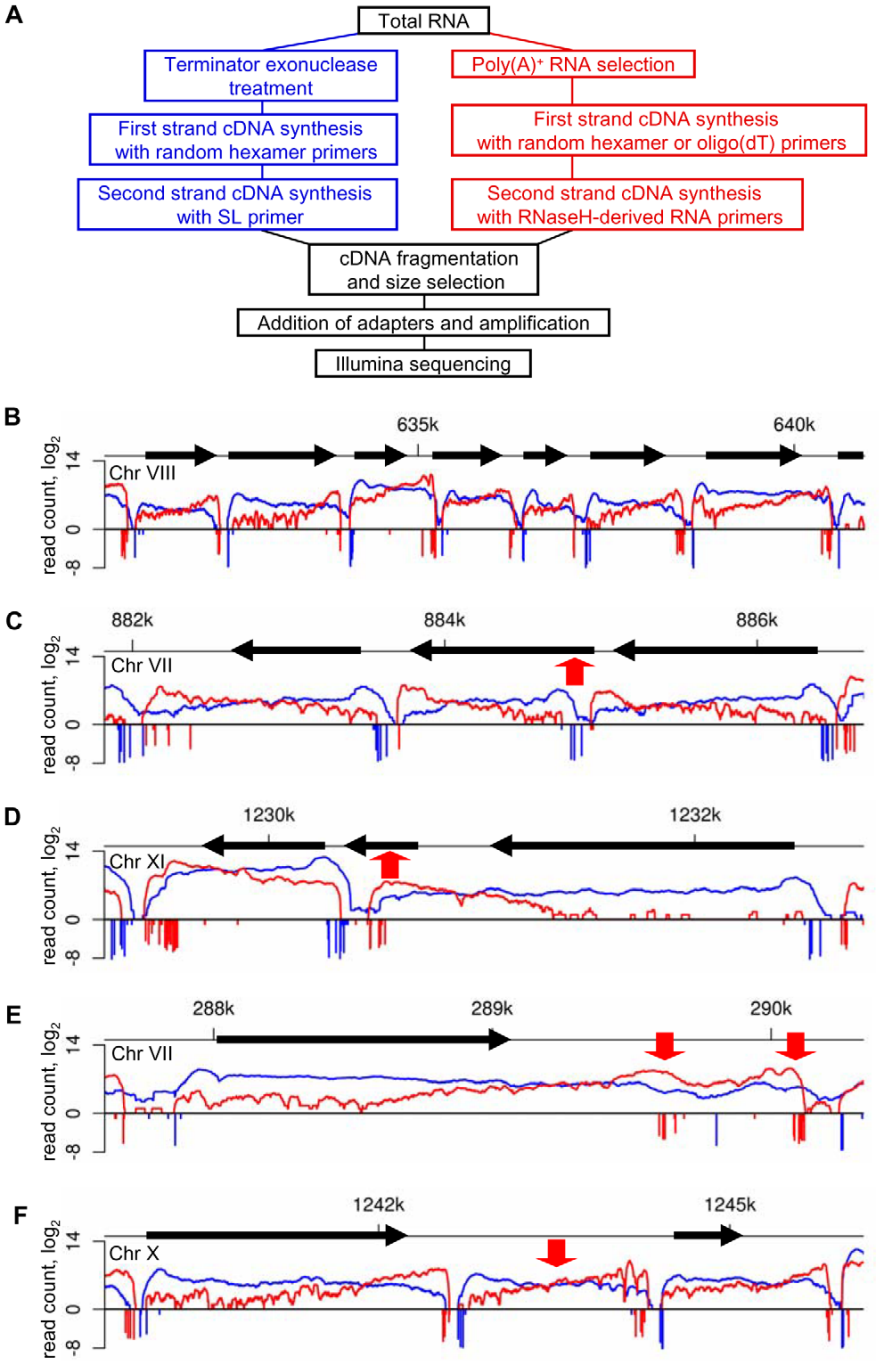
RNA-Seq precisely maps the ends of *T. brucei* transcripts. (A) Outline of the protocol for generating libraries for sequencing. (B) Overlay of the number of reads (log_2_) from 5′-end- (blue) and 3′-end- (red) enriched libraries aligning to ∼10 kb genomic region. Numbers of end-reads, SL-containing (blue) and poly(A)-containing (red), are shown below the x-axis (−log_2_). (C–F) Examples of misannotated start codons (C), genes not producing their own transcript (D), alternatively processed transcripts (E) and new transcripts (F). Red arrows point to the highlighted transcriptome features.

To map transcribed regions in *T. brucei* on a genome-wide scale, we used the RNA-Seq approach [18]. Starting with poly(A)^+^ RNA isolated from insect-form (culture-form adapted) *T. brucei rhodesiense* YTat 1.1 [19], double-stranded cDNA was generated conventionally with either random hexadeoxynucleotide or oligo(dT) primers (Figure 1A). We sequenced four libraries, namely two biological samples, each random-primed and oligo(dT)-primed (RNA-Seq data from this study have been submitted to the NCBI Sequence Read Archive - SRA at http://www.ncbi.nlm.nih.gov/Traces/sra/sra.cgi - under accession no. SRA012290). Pair-wise comparison of all sets resulted in Pearson correlation coefficients exceeding 0.99 (Figure S1), thus giving us a total of 30,860,548 sequence reads (Table 1). 25,245,618 reads (82%) aligned to the reference *T. brucei brucei* TREU 927 genome [1] by allowing up to two mismatches in the first 28 nucleotides (Table 1) with 16,651,856 reads (54%) mapping to a unique region in the genome. As expected based on similar approaches in yeast [20], this strategy resulted in an enrichment of RNA-Seq tags towards the 3′ end of transcripts (Figure 1B). To complement this bias, we took advantage of the unique structure of trypanosome mRNAs and devised a modified procedure for RNA-Seq. Briefly, total RNA was depleted of rRNAs by treatment with Terminator exonuclease, first-strand cDNA synthesis was performed with random hexamers and we then used the SL sequence present at the 5′ end of all trypanosome mRNAs to generate double-stranded cDNA with an SL-specific primer (Figure 1A). Three technical replicates were sequenced (Pearson correlation coefficients of 0.9) resulting in 33,338,202 total reads with 31,794,274 reads (95%) aligning to the genome (Table 1) with a clear preference for the 5′ end of transcripts (Figure 1B). Even though our RNA-Seq tags were obtained from a closely related *Trypanosoma* subspecies, overall 89% of the sequences aligned to the reference *T. brucei brucei* TREU 927 genome (Table 1). Furthermore, none of the tags aligned to the gene coding for the Tn10 transposase (Figure S2), which is present in the genome sequence assembly, but is the result of an artifact of BAC construction and thus is not a part of the genome [1].

**Table 1:** Statistics for RNA-Seq data sets.

5′ end-enriched reads	pcs1^a^	pcs2	pcs3	SL total
**Read length**	35	75	75	
**Total reads**	9,451,665	12,463,301	11,423,236	33,338,202
**Reads mapped by Bowtie**	9,087,899	11,847,276	10,859,099	31,794,274
**End-reads**	NA^b^	1,288,871	1,145,595	2,434,466
**Reads mapped to >1 location**	5,568,355	7,178,994	6,557,198	19,304,547
**Reads mapped to 1 location**	3,519,544	4,668,282	4,301,901	12,489,727
**Unmapped reads**	363,766	616,025	564,137	1,543,928

3′ end-enriched reads	pcr1^a^	pct1^a^	pcr2	pct2	pA total
**Read length**	35	35	35	35	
**Total reads**	7,645,925	7,837,447	7,188,204	8,188,972	30,860,548
**Reads mapped by Bowtie**	6,159,022	6,339,400	5,863,477	6,883,719	25,245,618
**End-reads**	97,754	70,003	82,830	78,294	328,881
**Reads mapped to >1 location**	2,094,805	2,138,252	2,010,506	2,350,199	8,593,762
**Reads mapped to 1 location**	4,064,217	4,201,148	3,852,971	4,533,520	16,651,856
**Unmapped reads**	1,486,903	1,498,047	1,324,727	1,305,253	5,614,930

a Pcs, pcr and pct indicate libraries from procyclic cells prepared with random primers, oligo(dT) primer and SL primer (for the second cDNA strand), respectively.

b Length of reads did not allow extraction of end-reads.

### Mining RNA-Seq data for gene boundaries and RNA levels

To map 5′- and 3′-end boundaries of trypanosome transcripts, we inspected the aligned data for regions of rapid changes in the abundance of RNA-Seq tags. In addition, our annotation was aided by the availability of end tags, i.e. 2,434,466 SL- and 328,881 poly(A)-containing reads (Table 1). This strategy allowed the precise mapping of both ends of 8,960 mRNA molecules with representative examples shown in Figure 1B. 898 previously annotated genes had very few reads and thus we were unable to precisely define their transcript boundaries. Most genes in this group are subtelomeric variant surface glycoprotein genes and pseudogenes (620), expression site-associated genes and pseudogenes (104), as well as unlikely ORFs, sequence orphans and conserved proteins (174). Our 5′-end mapping identified 532 genes (5.9% of all genes) with misannotated translation start codons (Figure 1C, Figure S3 and listed in Table S1) and RT-PCR experimentally confirmed that the ORFs of two genes on chromosome VIII were indeed shorter than currently annotated (Figure S3C). Furthermore, 805 annotated genes (not included in the total number of genes) did not produce a transcript of their own (Table S2), but were often part of the 5′UTR or 3′UTR of a transcript from a neighboring gene (Figure 1D and Figure S4), which we corroborated by Northern blot analysis for two such scenarios (Figure S4C).

We identified 441 genes that have unambiguous alternative processing in the 5′UTR (47 genes) or 3′UTR (394 genes), thus generating a full-length transcript, as well as a shorter ORF-containing and a putative non-coding transcript (Figures 1E and S5 and listed in Table S3). For instance, Tb927.4.4370 and Tb927.4.4490 fall into this category and by Northern blot analysis three distinct transcripts, as predicted by RNA-Seq, are easily detectable (Figure S5C). The functional significance of the UTR-internal transcripts remains to be investigated, since they have limited coding potential (Figure S6E) and it was inconclusive whether one such transcript derived from the 3′UTR of Tb927.4.4370 was associated with polyribosomes (Figure S6B).

The genome assembly of *T. brucei* lists four putative introns with two being experimentally validated [21], [22]. Using the program TopHat [23], which aims to identify splice junctions, and confirmed by manual inspection, we only detected the experimentally validated introns in the genes for poly(A) polymerase (Tb927.3.3160) and an ATP-dependent DEAD/H RNA helicase (Tb927.8.1510) and found no evidence for additional introns in the *T. brucei* genome (data not shown) in line with a similar conclusion by Siegel et al. [17].

Our data also shed light on the synthesis of annotated small nucleolar RNAs (snoRNAs), whose genes are always oriented in the same direction as neighboring protein coding genes and are known to be transcribed by Pol II [24], [25]. It appeared that snoRNAs are initially produced as long precursors with mRNA features, i.e. SL at the 5′ end and a poly(A) tail at the 3′ end, and these precursors may contain one or more snoRNA sequences. We noticed 27, 9 and 5 instances where snoRNAs mapped in 3′UTRs, predicted ORFs and 5′UTRs, respectively (Figure S7). Particularly intriguing is the scenario where 7 snoRNAs are embedded in the ORF of Tb927.3.1900 (Figure S7C), since these snoRNAs are expressed [25] and the predicted protein was detected in two separate proteomic analyses [26], [27]. This unusual organization will need to be investigated further.

**Figure 2:**
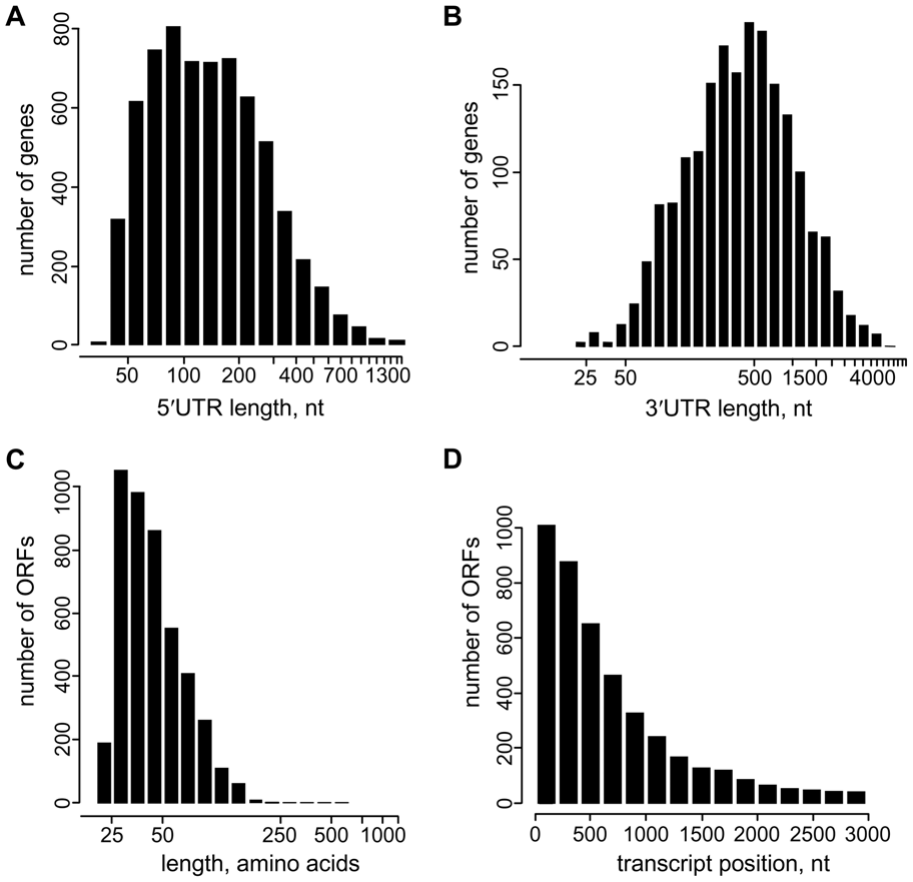
Characteristics of untranslated regions and novel transcripts in *T. brucei*. (A) Distribution of 5′UTR length (including the 39 nt SL), n = 6,577. (B) Distribution of 3′UTR length, n = 1,902. (C–D) Coding potential of novel *T. brucei* transcripts. (C) Length distribution of possible ORF-encoded polypeptides (nonoverlapping ORFs, equal or longer than 75 nt), n = 4,540. (D) Positional distribution of translation start sites for possible ORFs in novel transcripts, n = 4,540.

The above mapping of gene boundaries provided a platform to delineate and measure 5′ and 3′UTRs (Tables S4 and S5). The median length of 5′UTRs in *T. brucei* is 130 nt with a range from 39, with the SL abutting the initiation codon, to ∼2,500 nt (Figure 2A), whereas a similar analysis of 3′UTRs revealed a median length of 388 nt with a range of 10 to ∼6,000 nt (Figure 2B). The median lengths are in agreement with those described in Siegel et al. [17] of 128 nt and 400 nt for the 5′ and 3′UTRs, respectively. It is important to point out that in this study the 39-nt SL was included in the 5′UTR length calculation, whereas Siegel et al. opted to exclude the SL from their analysis [17].

Furthermore, by taking advantage of the quantitative nature of RNA-Seq [18], we were able to estimate RNA levels in the insect-form *T. brucei rhodesiense* YTat 1.1 strain (Table S6). We determined the signal in a 500-bp window immediately downstream of the first *trans*-splice site and, using the measured PGKB mRNA level as a reference point [28], we estimated that 75% of the genes generate between 1 and 10 mRNA molecules per cell (Figure S8). Our copy number estimates (median is 3 mRNAs per cell) for cultured procyclic *T. brucei* are comparable to data obtained for yeast [29], [30] and mammals [31].

### Novel coding and non-coding RNAs

We detected 1,114 new transcripts (12% of total) not originating from a previously annotated ORF (Table S7). They are *trans*-spliced and polyadenylated and the range of size and expression level closely resembles that of transcripts mapped to annotated ORFs (Figures 1F, 2 and S9 and Table S7). Setting a lower limit of 25 amino acids, 1,011 transcripts have the potential to encode one or more ORF with a considerable number having a predicted signal peptide (4.2%). In particular, 27 newly identified transcripts contain ORFs that are conserved and annotated in *T. cruzi* and/or *Leishmania major* (Table S8) and an additional 23 new transcripts encode potential polypeptides that are conserved, but not annotated in *T. cruzi* and/or *L. major* (Table S9). For instance, three novel transcripts on chromosome VIII, clearly detectable by Northern blot analysis, encode ribosomal protein L41 and two newly mapped transcripts on chromosome XI encode polypeptides that are conserved, but not annotated in *T. cruzi* and *L. major* (Figure S9). Tb10.NT.122 is a novel transcript on chromosome X with the longest predicted ORF encoding 32 amino acids. This transcript co-sedimented with polyribosomes on a sucrose density gradient and shifted to lighter fractions under conditions where translation was inhibited (Figure S6C), indicating a likely association with the translational apparatus. Finally, by searching the proteome data set of Panigrahi et al. that did not map to annotated genes [32], we identified 19 novel transcripts where the predicted ORF had one or more matches with peptides identified by mass spectrometry (Table S10), thus providing evidence that at least some of these new proteins are made.

Of the 1,114 novel transcripts, 9.2% (103) did not contain ORFs 25 amino acids or longer and ranged in size from 154 nt to 2,229 nt (Table S11). Although some of these transcripts might potentially code for a peptide, since the shortest verified coding sequence in eukaryotes is 33 nt, i.e. 11 amino acids [33], [34], it is worth noting that the six largest transcripts, each longer than 1,000 nt, have no coding potential at all (Table S11).

### Heterogeneity of RNA processing sites

**Figure 3:**
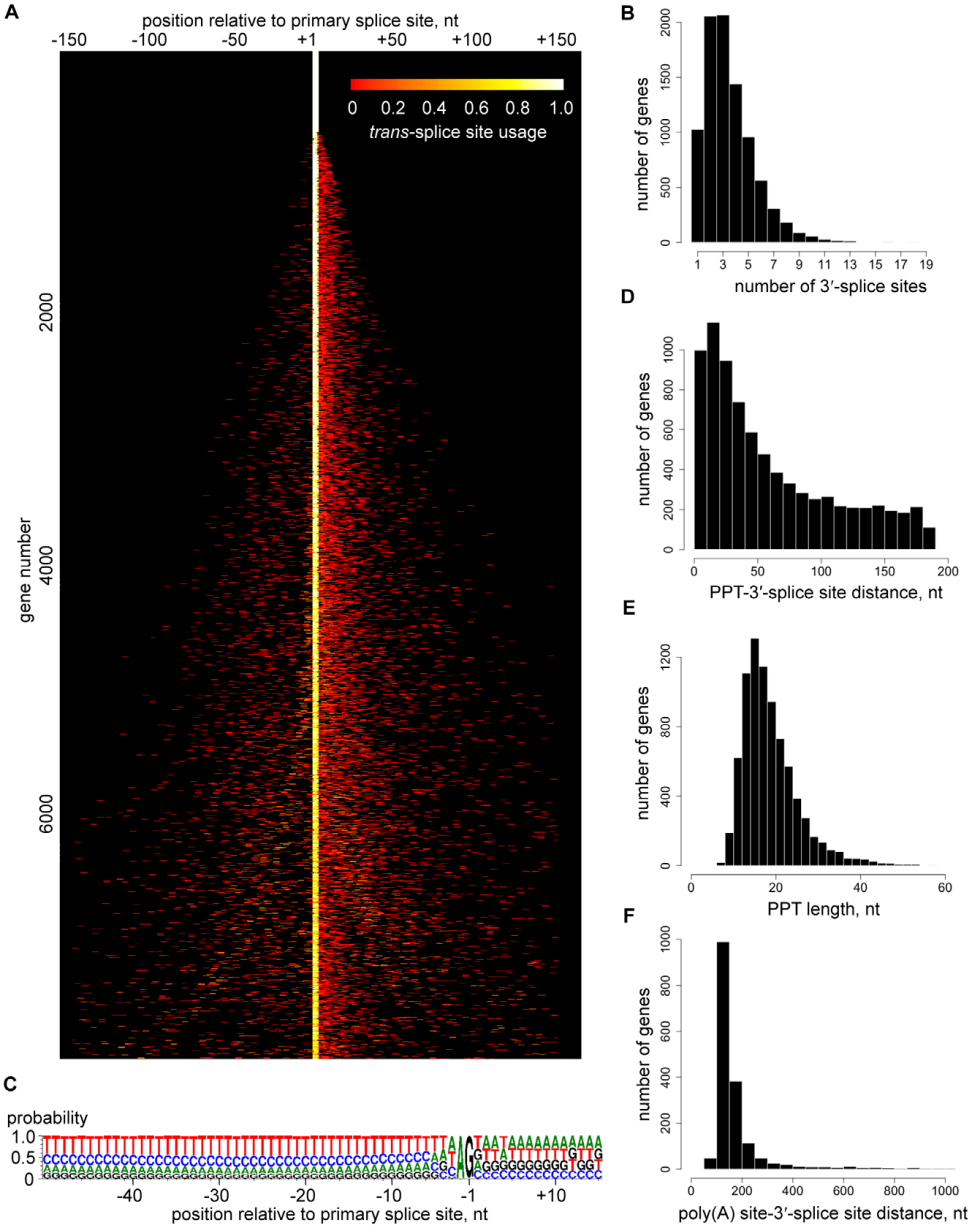
*Trans*-splice sites in *T. brucei*. (A) Heterogeneity of 3′-*trans*-splice sites. The *trans*-splice sites of each gene are shown in each line of the plot, n = 7,966. Each site is represented by a bar colored according to its usage (relative number of SL-containing reads). The site with the largest number of reads is centered, and sites within 150 nt upstream or downstream of this site are included. For display purposes, not all transcripts are shown. Genes are ranked from most homogeneous splice sites (top) to least homogeneous splice sites (bottom). (B) Number of *trans*-splice sites per gene, n = 8,815. (C) Compositional profile of the 3′-*trans*-splice site sequence, n = 7,966. Nucleotide positions are relative to the site of splicing. (D) Distribution of PPT–3′-*trans*-splice site distances, n = 7,996. (E) Distribution of PPT lengths, n = 7,966. (F) Distribution of poly(A) site–(downstream-)3′-*trans*-splice site distances, n = 1,759.

The depth of our coverage of 5′ end tags (Table 1) allowed us to expose a genome-wide picture of *trans*-splice sites, which turned out to be quite promiscuous (Figure 3A). Out of the 8,592 transcripts with at least 10 SL tags at the primary site, only 926 (11%) had a single site for SL addition, whereas 5,327 transcripts (62%) had between two and four 3′ *trans*-splice sites and 2,339 transcripts (27%) had five or more sites (Figures 3A and B). Additional sites were mainly located downstream of the primary site (Figure 3A) and occasionally mapped within the 5′ region of ORFs (Table S12). To validate this unexpected result, we first inspected *trans*-splice site usage at the α-tubulin gene (Figure S10). The most prominent site mapped by RNA-Seq coincided with the site identified by previous analysis of cDNAs, but minor sites were also evident. Next, we selected four genes representing the spectrum of heterogeneity and cDNAs generated by RT-PCR reproduced the multiple or highly homogenous SL addition sites seen by RNA-Seq (Figure S11).

We were unable to identify any significant difference in the sequence signatures surrounding homogeneous and heterogeneous splice sites (data not shown) and there was only a very limited correlation between splice site heterogeneity and abundance of the corresponding transcript (data not shown). The only highly conserved sequence signals that appeared to define a 3′-*trans*-splice site in *T. brucei* are the well-known AG dinucleotide immediately upstream and the polypyrimidine tract (PPT) further upstream of the splice site, as well as the exclusion of G residues at position -3 (Figure 3C), which has been suspected previously [12]. An AG dinucleotide was found at 98% of the major splice sites, whereas minor sites had an AG in 75% of the cases (Figure 3C and Table S4). At present we do not know whether this observation is of functional significance. Since two major sites with an apparent AA or GG dinucleotide in the 927 genome reference strain turned out to have *bona fide* AG splice acceptor sites in our strain used for RNA-Seq (data not shown), we cannot exclude the possibility that many of these variant non-AG sites are due to strain differences. The PPT starts 0 to ∼200 nt (median is 43) upstream of the splice site (Figures 3C and 3D), its median length is 18 nt with a range from 7 to 79 nt (Figure 3E) and its composition showed a clear preference for Ts over Cs (Figure 3C), in agreement with a comprehensive mutagenesis study [12]. The median distance between the *trans*-splice site and the upstream polyadenylation site is 142 nt and in 50% of the cases is between 123 and 178 nt (Figure 3F), highlighting the tight spatial coupling of the two RNA processing events [7], [8].

**Figure 4:**
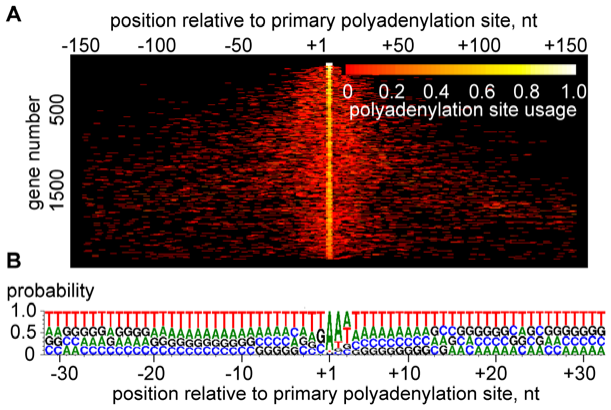
Heterogeneity of polyadenylation sites. (A) The polyadenylation sites of each gene are shown in each line of the plot, n = 2,081. Each site is represented by a bar colored according to its usage [relative number of poly(A)-containing reads]. The site with the largest number of reads is centered, and only sites within 150 nt upstream or downstream of this site are included. For display purposes, not all transcripts are shown. Genes are ranked from most homogeneous polyadenylation sites (top) to least homogeneous polyadenylation sites (bottom). (B) Compositional profile of *T. brucei* polyadenylation sites, n = 2,081.

Polyadenylation sites (PAS) in *T. brucei* transcripts appeared even more heterogeneous than *trans*-splice sites (Figure 4A and Table S13). A representative example is shown in Figure S10, where the processing sites in between the β and α-tubulin genes are summarized. The most prominent RNA-Seq sites correlated with experimental data [8], but this methodology also revealed an unanticipated extent of heterogeneity. The heterogeneity in 3′-end formation was not too surprising, since it is known that a poly(A) tail can be added at any one of several closely spaced positions [8], [13]. Polyadenylation occurs preferentially at an A residue (before or after; our data cannot distinguish) in the transcript that is often followed by a second A, and our results showed a preference for T-rich sequence in the vicinity of the site for poly(A) tail addition (Figure 4B).

### RNA-Seq tags map putative Pol II transcription initiation sites

**Figure 5:**
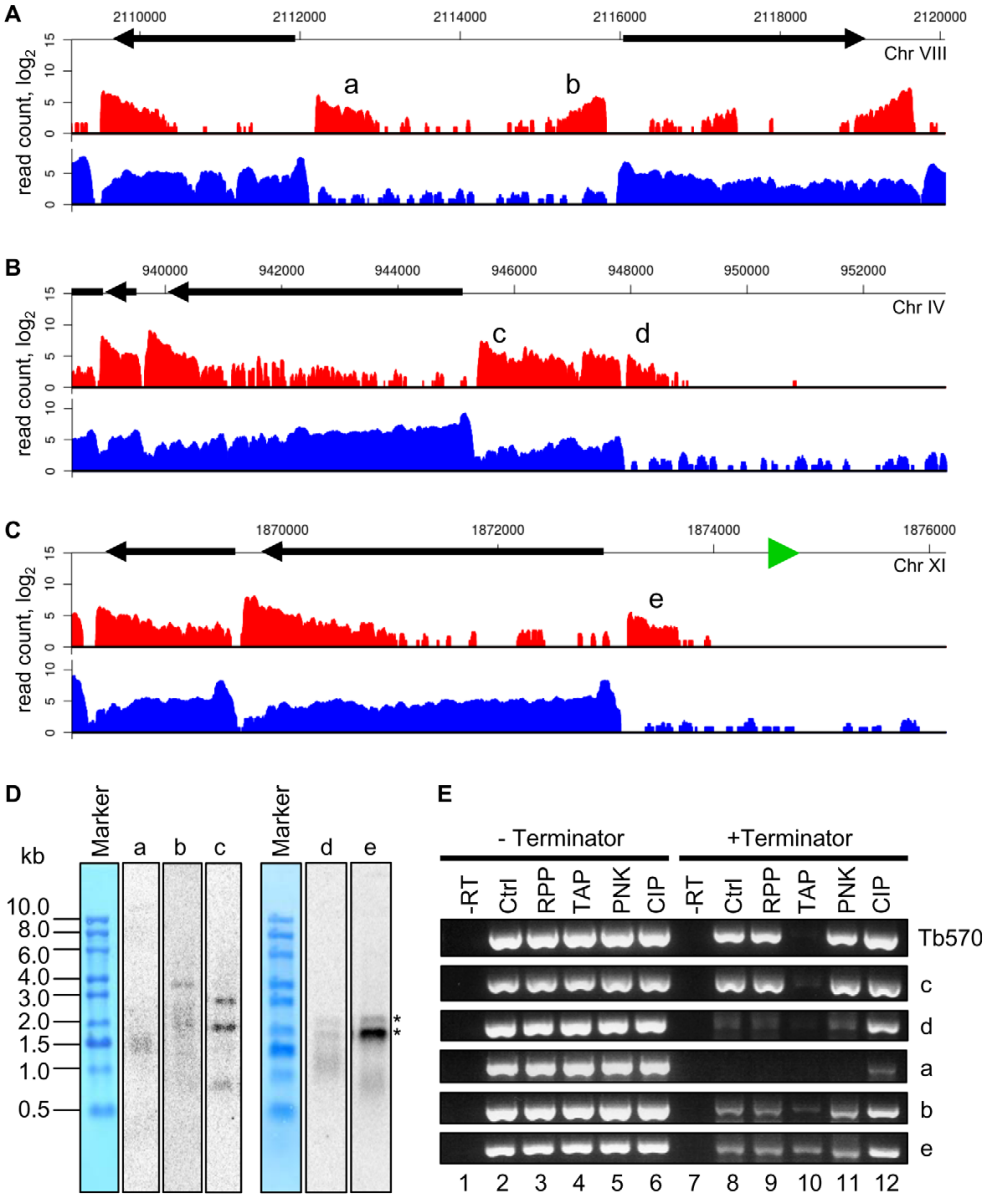
Polyadenylated transcripts without the SL sequence at suspected Pol II start sites. (A) Plots of the number of reads (log_2_) from poly(A)-enriched (red) libraries and SL-enriched (blue) libraries aligning to a region of divergent transcription. a, b – transcripts underrepresented in the SL-enriched libraries. (B) Plots of the number of reads aligning to a region adjacent to a putative centromeric region on chromosome IV. c – a novel transcript, d – transcript underrepresented in the SL-enriched libraries. (C) Plots of the number of reads aligning to a region adjacent to a tRNA gene (green triangle). e – transcript underrepresented in the SL-enriched libraries. (D) Northern blots of RNA after one round of oligo(dT) selection with probes against the indicated transcripts. RNA size marker is visualized with methylene blue staining of the membrane. Multiple sizes transcripts are present for b and c, as suggested by the reads alignment plots in (A) and (B). Asterisks indicate bands cross-hybridizing to rRNA for the d and e blots. (E) RT-PCR assay to determine the nature of the 5′ ends of transcripts. After incubation of total RNA with the indicated enzymes, the samples were treated with or without Terminator exonuclease prior to reverse transcription and PCR. –RT – control sample without reverse transcriptase; Ctrl – control sample treated without any 5′-end-modifying enzyme; RPP – RNA 5′ polyphosphatase; TAP – tobacco acid pyrophosphatase; PNK – T4 polynucleotide kinase; CIP – calf intestinal alkaline phosphatase. Tb570 is a control detecting mRNA for Tb10.6k15.1610.

An intriguing observation was the presence of a limited number of polyadenylated transcripts that appeared to be significantly underrepresented or even absent in our SL library data sets. In particular, these transcripts mapped closely to regions in the genome previously identified as putative Pol II transcription initiation sites [4], [35], [36], namely strand-switch regions of divergent transcription units, the beginning of Pol II transcription units that neighbor tRNA genes, shown previously to be strong stops for Pol II [37], as well as a few other regions within transcription units (Figures 5A, B and C). Such transcripts can be detected as heterogeneous species by Northern blotting (Figure 5D) and a large fraction of them appeared to be uncapped and have a single phosphate at their 5′ end, as judged by their susceptibility to degradation by Terminator exonuclease (a 5′ monophosphate-dependent enzyme) and alleviation of this susceptibility by a preceding phosphatase treatment (compare lanes 2, 8 and 12 in Figure 5E). In contrast, at most suspected ends of Pol II transcription units, we detected transcripts of extremely low abundance, i.e. less than one molecule per cell, that possess a SL sequence, but lack poly(A) tails, as judged by their absence in our poly(A)-enriched libraries (data not shown). [The transcripts without SL sequence or a poly(A) tail were not included in the total number of genes or in the number of novel transcripts.] Transcripts present near putative Pol II start sites lacking the SL but containing a poly(A) tail, as well as those with an SL but missing the poly(A) tail at possible Pol II termination sites, are best explained by the known coupling of *trans*-splicing and polyadenylation [7], [8]. For example, *trans*-splicing of the first mRNA in a polycistronic transcript will by default result in the polyadenylation of the upstream RNA and thus generate the transcripts we detected.

**Figure 6:**
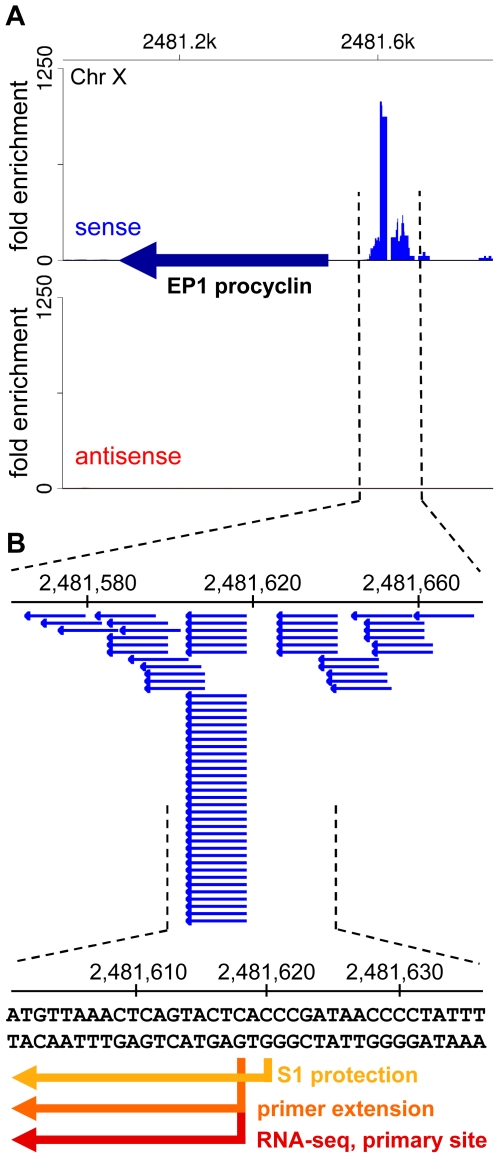
A library enriched for 5′-triphosphate ends accurately captures *bona fide* transcription start sites. (A) Shown is a segment of chromosome X surrounding the EP1 procyclin gene. The EP1 ORF is indicated by a dark blue arrow. The fold enrichment of reads from the 5′-triphosphate end library [(number of reads in the 5′-triphosphate end-enriched library)×24/(number of reads in the 5′-end enriched library)] are plotted for the plus strand (sense, red) and the minus strand (antisense, blue). (B) Shown is the region surrounding the transcription start site for RNA polymerase I with the individual aligned reads (blue horizontal arrows). Since this library was prepared after oligo(dT) selection of transcripts, the obtained reads are most likely derived from the extremely low-abundance, full-length polyadenylated EP1 procyclin transcripts that have not been *trans*-spliced. Indicated by arrows in different shades of orange-red are previously determined transcription start sites by S1 protection [52] and primer extension [53], as well as the primary start site identified by RNA-Seq (this study).

**Figure 7:**
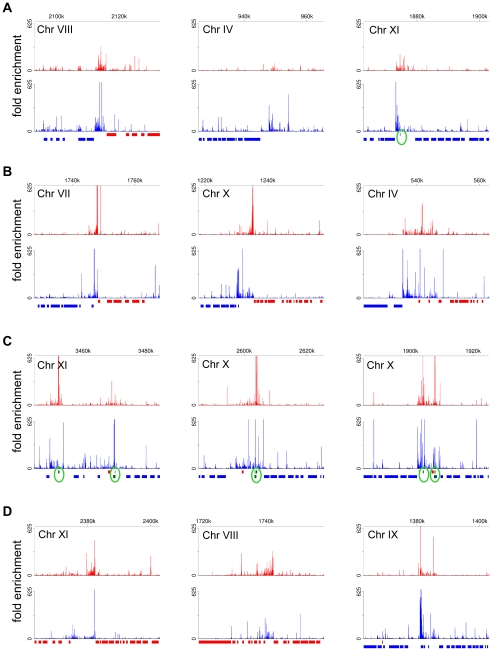
Putative Pol II initiation sites in *T. brucei*. Plots for the fold enrichment of reads from the 5′-triphosphate end library [(number of reads in the 5′-triphosphate end-enriched library)×24/(number of reads in the 5′-end enriched library)] are shown aligning to ∼40 kb genomic region for the plus strand (red) and the minus strand (blue). Annotated ORFs are shown in colored bars corresponding to their orientation. Annotated tRNAs are shown as green bars and are highlighted with green circles. (A) Plots for the examples shown in Figure 5A–C. (B) Examples of regions of divergent transcription. (C) Examples of genomic loci in proximity to tRNA genes. (D) Examples of genomic regions within transcription units.

The SL-lacking RNAs described above could represent remnants of primary Pol II transcripts, i.e. RNA fragments from the very 5′ end of long polycistronic precursors that have either been processed by an unknown mechanism to expose a 5′-monophosphate or have been subjected to trimming of their triphosphate ends to a 5′-monophosphate. To explore this possibility, we generated and sequenced a cDNA library enriched for 5′-triphosphate RNA ends, the hallmark of a 5′ end generated by an RNA polymerase (Figure S12). The validity of our protocol was underscored by correctly mapping the transcription initiation site of the EP1 procyclin gene (Figure 6). Corroborating our hypothesis, RNA-Seq tags were again enriched in regions of the genome implicated to be sites for transcription initiation by Pol II (Figure 7A). In particular, this enrichment was observed at all sites suspected to act as Pol II promoters based on the accumulation of specifically modified and variant histones [36], i.e. primarily at every divergent transcription unit (Figure 7B and Table S14), at regions located at the beginning of transcription units in proximity to tRNA genes (Figure 7C and Table S14), as well as at internal sites within transcription units (Figure 7D and Table S14). One common theme of all regions was that they showed enrichment of not only sense, but also antisense 5′-triphosphate RNA-derived reads (in contrast to a Pol I transcription unit, Figure 6), suggesting that these putative Pol II transcription initiation sites are intrinsically bi-directional.

## Discussion

The current annotation of the *T. brucei* genome relied heavily on bioinformatic approaches, since to date a mere 5,133 ESTs are available. Thus, our initial goal in the work presented here was to map gene boundaries at the transcript level and to provide experimental support for annotated features. We used RNA-Seq to generate a *T. brucei* transcriptome map at single-nucleotide resolution and confirmed many of the gene models, but also uncovered numerous cases of misannotated translation initiation codons, annotated ORFs not producing a distinct transcript and an abundance of novel transcripts, including ones which appear to be non-coding. Remarkably, we were also able to map, on a genome-wide scale, putative Pol II transcription start sites in *T. brucei* (summarized in Table S14). Our results corroborate a recent study where it was shown that such chromosome locations are marked by specifically modified and variant histones [35], [36]. In addition, we detected both sense and antisense transcription originating from these genome loci, with sense transcripts at higher levels than antisense (Figure 7). This finding matches a scenario described for mammalian CpG promoters [38]–[40], where transcription is divergent and initiates over a broad genomic region. Moreover, our RNA-Seq data for 5′-triphosphate-enriched RNAs showed reads mapping throughout Pol II transcription units in the sense and antisense orientation (Figure S13) and thus, we cannot exclude the possibility that these are actual transcription initiation sites similar to the ones described in exons/3′ UTRs in mammals [38].

**Figure 8:**
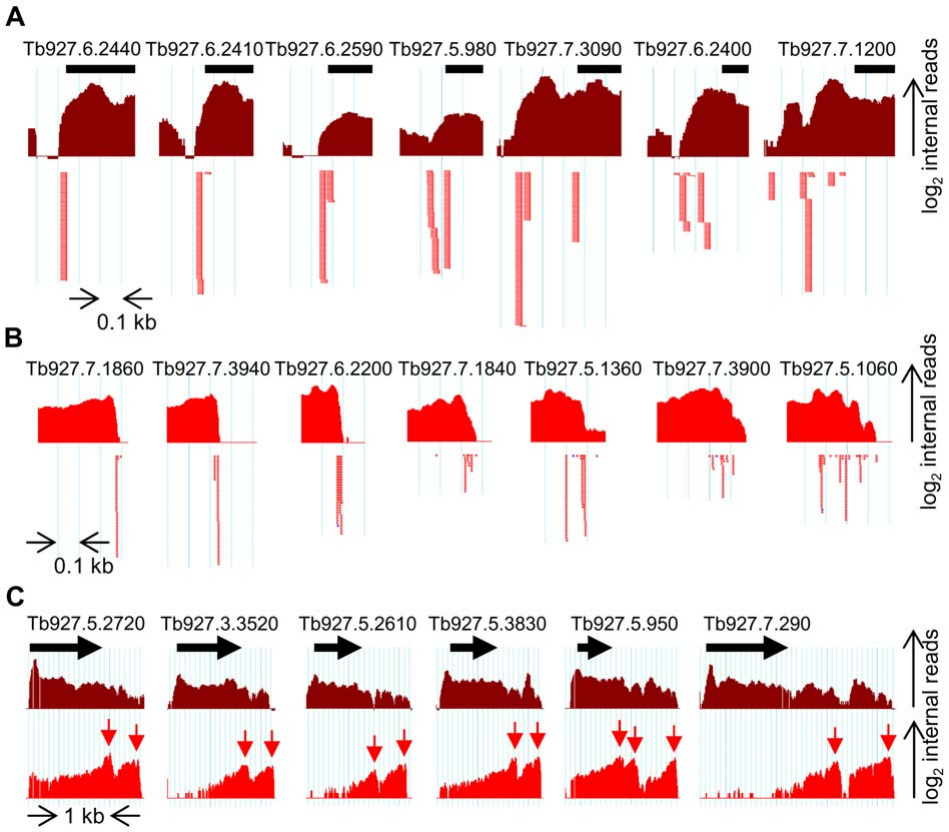
Complexity of pre-mRNA processing patterns in *T. brucei*. (A) Examples of homogeneous (left) and heterogeneous (right) *trans*-splice sites. Shown is the pileup of number of internal reads (log_2_) from 5′-end-enriched libraries (dark red). Individual SL-containing reads are depicted by red arrows under the graph. The identity of each gene is indicated above the black bar representing the beginning of the ORF. The light blue vertical lines are 100 nt apart. (B) Examples of homogeneous (left) and heterogeneous (right) polyadenylation sites. The pileup of number of internal reads (log_2_) from 3′-end-enriched libraries (red) is depicted above the individual poly(A)-containing reads (red dots). Scale as in (*A*). (C) Examples of “truly” alternatively processed transcripts. The number of internal reads (log_2_) from 5′-end- (dark red) and 3′-end-enriched (red) libraries aligning to the genomic regions surrounding the indicated ORFs (black arrows) are depicted. Easily identifiable peaks in the 3′-end-enriched libraries pileup (red arrows) and end-reads (not shown) indicate the alternative 3′ ends of the transcripts containing the ORFs. Note the scale difference from (A) and (B).

One of the major benefits of RNA-Seq is its capability to accurately determine transcript boundaries. Our mapping of conventional 5′ and 3′ ends of *T. brucei* mRNAs revealed an abundant heterogeneity in *trans*-splice and polyadenylation sites. Whereas the heterogeneity of poly(A)-addition sites was somewhat anticipated [8], [13] and to a lesser extent this scenario has been observed as local heterogeneity of polyadenylation positions in yeast [20] and mammals [41], [42], the degree of variability we encountered for *trans*-splice sites was quite unexpected. Although *trans*- and *cis*-splicing are mechanistically related, our finding highlights a fundamental difference between these two processes. *Cis*-splicing occurs predominantly within ORFs [43] and, thus, is subjected to evolutionary pressure to maintain an intact coding sequence. This apparent accuracy of intron excision is likely accompanied by rapid destruction of aberrantly spliced mRNAs by the nonsense-mediated decay pathway [44]. In contrast, *trans*-splicing mainly takes place upstream of ORFs and appears to follow more relaxed rules for the selection of a nucleotide for the second *trans*-esterification reaction of splicing. As a consequence, the observed heterogeneity in *T. brucei* of both *trans*-splicing and polyadenylation generates a collection of mRNAs with the same coding potential, but with UTRs (3′ or 5′) of varying length, sometimes in the order of hundreds of nucleotides. This fluidity in generating mRNA ends is quite unusual, particularly in an organism where the regulation of gene expression has been attributed to occur mainly at the post-transcriptional level [3], [6]. Importantly, the degree of heterogeneity of both *trans*-splicing and polyadenylation varies and different genes exhibit RNA processing patterns spanning from one extreme - a single detectable processing site - to the other, where no single site is used preferentially (Figure 8).

Overall, our data set is in good agreement with that of Siegel et al. [17]. The overlap between the mapped *trans*-splice sites is excellent with most genes having a very high correlation (Figure S14A). There is a weak overlap between the polyadenylation sites (Figure S14B). This is most likely a reflection of the inherent difficulty to identify PAS due to the pervasive heterogeneity (Figure 4) and the prevalence of low-complexity sequences in the 3′UTR. Thus, our stringent filtering only yielded 2,081 PAS assignments with confidence (Figure 4). Finally, the mRNA abundance comparison (for the 6,728 transcripts present in both studies) is good with a Pearson correlation coefficient of 0.697 (Figure S15), considering the different *T. brucei* subspecies, as well as differences in culture conditions, mRNA isolation procedures, RNA-Seq cDNA library preparation protocols and methods of estimating the abundance.

Evidence and theorization over the past decade has resulted in a widespread appreciation of the benefits of stochastic models of gene expression over purely deterministic ones [45]–[47]. We estimate that 75% of *T. brucei* genes are expressed at levels between 1 and 10 mRNAs per procyclic cell (Figure S8). In addition, it is evident from our data and corroborated by Siegel et al. [17] that the majority of genes produce different transcript forms created by heterogeneity in *trans*-splicing and polyadenylation and bearing or lacking 5′ and/or 3′ UTR sequences that could influence the efficiency of RNA stability and/or translation. The possibility for regulation of pre-mRNA processing site choice clearly exists, however it remains to be seen whether alternative splicing or polyadenylation are part of a regulated process. Since transcription is one of the main intrinsic noise sources [47] and Pol II transcription in trypanosomatids appears to be uniform across individual polycistronic transcription units (and likely the entire Pol II transcriptome), heterogeneity in the sites for pre-mRNA processing provides an additional source of intrinsic stochasticity to compensate for the uniformity in transcription. Interestingly, it appears that heterogeneity of *trans*-splicing is a specific characteristic of trypanosomatids, since it was not described in the transcriptome analysis of the nematode *Caenorhabditis elegans* [48], an organism with prevalent *trans*-splicing in which most *trans*-spliced genes are individually transcribed [49]. Our results have implications for gene expression patterns in other important human pathogens and point to heterogeneity in pre-mRNA processing (rather than transcription) as the main intrinsic source of stochasticity in gene expression for these protozoan parasites.

## Methods

### 3′ end-enriched libraries

Total RNA was treated with RQ1 RNase-free DNase I and subjected to two rounds of poly(A)^+^ selection. First strand cDNA synthesis was initiated with random hexadeoxynucleotide primers or 5′-T_15_VN-3′ oligonucleotide. After incubation with RNase H and *E. coli* DNA polymerase I, double-stranded cDNA was fragmented with DNase I and cDNA fragments corresponding in size to about 200 bp were size-selected on an agarose gel. The cDNA ends were repaired, a single dA was added at the 3′ ends and genomic adapters (Illumina, Inc. All rights reserved.) were added. Libraries were enriched by limited PCR and purified on an agarose gel.

### 5′ end-enriched libraries

Total RNA was treated with Terminator 5′-monophosphate-dependent exonuclease, followed by DNase I and first strand cDNA synthesis was initiated with random primers. Second strand cDNA synthesis was primed with the SL Primer (5′-GCTATTATTAGAACAGTTTCTGTACTATATTG-3′) and platinum Pfx DNA polymerase. cDNA was further processed as described above.

### 5′-triphosphate-end-enriched libraries

Total RNA was subjected to two rounds of poly(A)^+^ selection and treated with Terminator 5′-phosphate-dependent exonuclease. Next, the RNA was treated with RNA 5′-polyphosphatase and a 5′ adapter with a BpuE I restriction site (5′-GCACCATATAACCGCTTCCrUrUrGrArG-3′) was ligated to the available 5′-monophosphate ends. Following first strand cDNA synthesis as described above, double-starnded cDNA was generated with a BpuE I Primer (5′-GCACCATATAACCGCTTCCTTGAG-3′) and Pfx DNA polymerase. DNA fragments larger than 100 bp were gel purified, digested with BpuE I in the presence of *S*-adenosylmethionine. The DNA was separated on an agarose gel and several gel segments spanning the ∼130 bp size range were excised and processed for Illumina sequencing.

### Enzyme assays for RNA 5′-end analysis

Reactions with 5′-end-modifying enzymes were performed in a volume of 40 µL with 14 µg total RNA with 40 U RNA 5′-polyphosphatase, 40 U tobacco acid pyrophosphatase, 10 U T4 polynucleotide kinase plus 1 mM ATP, or 1 U alkaline phosphatase. After the appropriate incubation, reactions were divided into two tubes and one aliquot was treated with 1 U Terminator 5′-phosphate-dependent exonuclease and the second aliquot served as a control. Reverse transcription with random hexamers was performed and the resulting cDNA was used as a template for PCR with forward and reverse primers specific for the transcripts of interest.

### Processing of 5′ end reads

Over 2.5 million reads from the SL-primed library contained the entire splice-leader sequence at their 5′ ends. This sequence was removed, leaving 43 nucleotides, and the first 28 nucleotides were aligned to the genome reference, with a maximum of 2 allowed mismatches and one alignment reported per read. Unless indicated, all read manipulations, as well as the genome-wide site analyses, were performed with custom scripts written either in Perl or for a combination of the R statistical software with the bioinformatics-centric Bioconductor tools installed.

### Processing of 3′ end reads

Reads consisting primarily of A or T nucleotides (more than 31 out of 35 total nucleotides) were removed from the 3′ end-enriched libraries. Next, contiguous A or T stretches, 5–15 letters long, were trimmed off the 5′ or 3′ ends of the sequences, respectively. Trimmed reads, ranging in length from 15–30 nucleotides, represented putative 3′ end-reads and were aligned to the genome reference. In order to distinguish poly(A) tails from RNA transcribed from contiguous As in the genome, alignments were only considered to represent polyadenylation sites, if the end-read contained a longer stretch of A's than was present in the genome.

### Analysis of 5′-triphosphate-end-enriched libraries

Preparation of the 5′-triphosphate-end-enriched library produced two sets of reads, categorized by the position of the 14-nt sequence representing the 5′ transcript end (the “end-sequence”). The first set, corresponding to the top strand contained the 24 nt adapter (GCACCATATAACCGCTTCCTTGAG) at the beginning of the read, followed by the end-sequence, spanning nucleotides 25 through 38. The second set contained reverse complement of the end-sequence in the left-most 14 nt, followed by the same reverse complement of the 24 nt adapter. These 14 nt end sequences were extracted from the two sets of reads, and the second set was reverse complemented, to produce the end-reads. Alignment to the genome was with no mismatches allowed, and one alignment reported per read.

### Alignment and interactive analysis of RNA-Seq data

Sequence reads obtained from the Illumina GA2 platform, ranging from 35–75 nucleotides in length, were aligned to the eleven major chromosomes in the *T. brucei* genome sequence, version 4 (genedb.org). All alignments were conducted with Bowtie [50]. Alignments were only considered if they contained fewer than two mismatches in the first 28 nucleotides of the alignment. For transcript-end analyses, end-reads were first identified then trimmed before alignment. Reads reporting multiple alignments were retained and aligned pseudo-randomly (according to Bowtie's default method). Processed RNA-Seq reads, including end-tags, were loaded into a customized, local installation of the Generic Genome Browser [51] for interactive annotation and analysis of transcript ends and novel transcriptome features.

### Measurement of transcript abundance

A relative measure of transcript abundance was derived from the number of reads (not including end-reads) that aligned within a 500-nucleotide window, extending into the gene from the 5′ end of the transcript for the SL-library and from the 3′ end for the poly(A) library.

### Polypyrimidine tract calculations

The polypyrimidine tract was identified as the longest stretch of pyrimidines separated by no more than one purine in a 200 nt window upstream of the splice site with the maximal number of reads.

### Transcript-end calculations

We eliminated from the analysis transcripts in which the maximal number of reads per splice site was less than 10. We developed a metric, *dispersion*, to capture and summarize the positional information about the splice sites of each gene. Essentially, dispersion is the weighted average of the distance between each site to the most popular site:

Assume a gene has N splice sites. Define *c_i_* as the number of reads of site *i* and *r_i_* as the position of the site. Denote 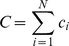 and let i^*^ represent the splice site with the maximal number of reads. The splicing dispersion is then defined as:
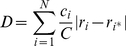

Similarly, the average 5′ UTR length is the weighted average of the distance of all splice sites from the start codon. Symbolically:

if the position of the start codon is r_s_, the average 5′UTR is:
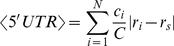

For UTR analyses, except where noted, splice sites downstream of the annotated start codon are not included in the sum (and C is the sum only over splice sites upstream of the start codon). Misannotated or unannotated transcripts were not considered for this analysis. We used a similar approach for analysis of polyadenylation sites, 3′UTRs.

Additional methods can be found in Supporting Information S1.

## Supporting Information

Figure S1 Comparison of technical and biological replica data sets. (A–I) Transcript abundance measures were derived from the number of reads (not including end-reads) that align within a 500-nucleotide window at the 5′ end of the transcript (for the SL-libraries) or at the 3′ end (for the poly-A enriched libraries; random primed or oligo(dT) primed). Shown above each graph are the Pearson's correlation coefficient (ρ_P_) and the Spearman's rank correlation coefficient (ρ_S_). Pcr, pct and pcs indicate libraries from procyclic cells prepared with random primers, oligo(dT) primer and SL primer (for the second cDNA strand), respectively.
(0.64 MB PDF)

Figure S2 Accuracy of alignment of sequence reads to the *T. brucei* reference genome. Shown is the overlay of the number of reads (log_2_) from 5′-end- (blue) and 3′-end-enriched (red) libraries aligning to ∼4kb window on chromosome III that contains the ORF for Tn10 transposase (Tb927.3.1050). Numbers of end-reads [SL-containing, blue; poly(A)-containing, red] are also shown (−log_2_). ORFs are represented by black arrows. No reads align to the Tn10 transposase ORF, which clearly is inserted within the sequence of a single transcript that covers both Tb927.3.1040 and Tb927.3.1060 (both currently annotated as interrupted, conserved hypothetical protein pseudogenes). According to GeneDB (www.genedb.org), the Tn10 insertion is an artifact from BAC construction and is not present in the *Trypanosoma brucei brucei* strain 927 genomic DNA or other BACs covering this region [1].
(0.07 MB PDF)

Figure S3 Experimental validation of misannotated translation start codons in *T. brucei* genes. Overlay of the number of reads (log_2_) from 5′-end- (blue) and 3′-end- (red) enriched libraries aligning to the shown regions of chromosome VIII covering the ORFs for Tb927.8.1270 (A) and Tb927.8.2000 (B). Numbers of end-reads (−log_2_) are also shown [SL, blue; poly(A), red]. Dashed lines indicate the positions of a gene-specific primer, the newly annotated *trans*-splice site and the currently annotated ATG for each of the two genes. Green bars indicate the potential products from an RT-PCR assay with SL and gene-specific primers. (C) RT-PCR assay. Poly(A)^+^ RNA was reverse transcribed with random primers and the resulting cDNA was used as a template for nested PCR with an identical SL forward primer for both amplification steps. Nested PCR was used to ensure specificity of amplification since the forward SL primer anneals to cDNA products from all *T. brucei* mRNAs. The sizes of the amplified products indicate that the ORFs for the corresponding genes are shorter than currently annotated.
(0.13 MB PDF)

Figure S4 Experimental validation of misannotated *T. brucei* genes that are part of transcripts from neighboring genes. Overlay of the number of reads (log_2_) from 5′-end- (blue) and 3′-end-enriched (red) libraries aligning to the shown regions of chromosomes VIII (A) and I (B). Black arrows represent ORFs encoding conserved proteins and grey arrows represent ORFs for a sequence orphan Tb927.8.5790 (A) and the unlikely hypothetical proteins Tb927.1.3060, Tb927.1.3080, Tb927.1.3090 and Tb927.1.3100 (B). (C) Northern blots of total RNA fractionated on denaturing agarose gels with probes against the indicated ORFs (adjacent lanes of the gel were used for the blots with different probes). The probes against Tb927.8.5780 and Tb927.8.5790 are detecting the same size transcript of ∼2.9 kb [∼2.8 kb plus a poly(A) tail]. The probes against Tb927.1.3070 and Tb927.1.3100 are also detecting the same size transcript of ∼2.6 kb [∼2.5 kb plus a poly(A) tail]. Positions of markers are indicated on the left.
(0.13 MB PDF)

Figure S5 Experimental validation of *T. brucei* genes that produce alternatively processed transcripts. Overlay of the number of reads (log_2_) from 5′-end- (blue) and 3′-end-enriched (red) libraries aligning to the shown regions of chromosome IV covering the transcripts produced for Tb927.4.4370 (A) and Tb927.4.4490 (B). Black arrows represent the annotated ORFs and purple arrows represent transcripts suggested by the internal tags (peaks in the pileups are indicated with red and blue downward arrows) and end-reads (not shown) alignment to the *T. brucei* genome. Note that Tb927.4.4370 is another example of a gene with a misanotated translation start codon. The positions of the regions hybridizing to specific probes are indicated by short black lines. (C) Northern blots of total RNA fractionated on denaturing agarose gels with the indicated (a–d) probes. Both probes a and b detect the full-length Tb927.4.4370 transcript. Probe a additionally detects a shorter transcript containing the Tb927.4.4370 ORF, while probe b additionally detects a shorter transcript that is a part of the 3′ UTR of the full-length Tb927.4.4370 transcript. Probes c and d both detect the full-length Tb927.4.4490 transcript. Probe c additionally detects a shorter transcript containing the Tb927.4.4490 ORF, while probe d additionally detects a shorter transcript that is a part of the 3′ UTR of the full-length Tb927.4.4490 transcript. Positions of marker RNA bands are indicated on the left.
(0.17 MB PDF)

Figure S6 A novel transcript with limited coding potential is possibly associated with polyribosomes. (A) Cell extracts prepared in the presence of pactamycin (an antibiotic that promotes apparent polyribosome dissociation through inhibition of the formation of complete translation initiation complexes) or cycloheximide (an antibiotic that arrests polyribosomes by interfering with ribosome translocation) were subjected to sucrose gradient ultracentrifugation. RNA was purified from individual gradient fractions, separated on a denaturing agarose gel, transferred and cross-linked to a nylon membrane, and large rRNAs were visualized by staining with methylene blue. Shown is every other fraction from the gradient. (B) Northern blot of the fractionated RNA with a probe detecting the full-length Tb927.4.4370 transcript (FL) and a shorter transcript that is a part of the 3′UTR of the full-length Tb927.4.4370 transcript (3′UTR) as a result of alternative processing (probe b in Supplemental Fig. S5). (C) Northern blot with a probe detecting a transcript from a novel gene on chromosome X (Tb10.NT.122). (D) Northern blot with a probe against α-tubulin transcripts. Note the shift of α-tubulin mRNA from polysome fractions of the gradient in the cycloheximide-treated extract to lighter gradient fractions in the pactamycin-treated extract. A similar shift is seen for Tb10.NT.122 (C). Three frame translation for Tb927.4.4370 3′UTR transcript (E) and Tb10.NT.122 (F) highlighting the limited coding potential of these transcripts. All ORFs are colored orange.
(0.20 MB PDF)

Figure S7 Examples of snoRNA-precursor transcripts. (A) A transcript containing a single snoRNA. (B) Multiple snoRNAs embedded in the 3′ UTR of a protein coding transcript. (C) Multiple snoRNAs embedded in the ORF of a protein coding transcript. (D) A snoRNA cluster producing multiple precursor transcripts containing more than one snoRNA. All panels show the overlay of the number of reads (log_2_) from 5′-end- (blue) and 3′-end-enriched (red) libraries. Numbers of end-reads (−log_2_) are also shown (SL, blue; poly(A), red). Black arrows represent currently annotated ORFs and red arrowheads represent mature snoRNA sequences.
(0.17 MB PDF)

Figure S8 Abundance profile of *T. brucei* transcripts. Relative transcript abundance represents the number of reads (not including end-reads) that align within a 500-nucleotide window at the 5′ end of the transcript (combined for the SL-library replicas) for each gene. Plotted is the number of genes with identical number of reads aligning to the 500 nt window. Genes without reads aligning to their sequence are not shown. Calculation of the estimated number of mRNA molecules per cell was based on using the precisely measured PGKB mRNA level in cultured procyclic *T. brucei* cells [28] as a reference point. Our estimates for mRNAs copy numbers in *T. brucei* closely resemble data obtained for yeast [29], [30] and mammals [31]. Darker shades of red background indicate higher copy numbers per cell.
(0.04 MB PDF)

Figure S9 Experimental validation of transcripts for novel *T. brucei* genes. (A) Overlay of the number of reads (log_2_) from 5′-end- (blue) and 3′-end-enriched (red) libraries aligning to the shown regions of chromosomes VIII and XI. Transcripts for novel genes (purple bars) are labeled a through e and their approximate sizes indicated [excluding the poly(A) tail]. (B) Sequence of the polypeptides encoded by putative ORFs in the indicated (a–e) transcripts. a, b, and c encode the *T. brucei* ribosomal protein L41. Also shown are the sequences for the three *Leishmania braziliensis* L41 polypeptides (encoded by three unannotated, tandemly arranged genes) and the human L41 sequence. Identical amino acids between the sequences from the three species are colored red. d and e encode 56 AA and 62 AA proteins respectively that are conserved but not annotated in *L. braziliensis*. (C) Northern blots of total RNA fractionated on denaturing agarose gels with probes against the indicated (a–e) transcripts. The (a, b, c) blot was performed with a probe against the short ORF present in all three transcripts. Transcripts a and b have almost identical size and they co-migrate during electrophoretic separation of the RNA sample. Positions of marker RNA bands are indicated on the left.
(0.17 MB PDF)

Figure S10 Comparison between RNA-Seq mapped processing sites with sites mapped by previous sequencing of cDNA clones [8]. Polyadenylation sites and 3′-*trans*-splice sites mapped by cDNA clones sequencing in the β-tubulin/α-tubulin inter-ORF region are indicated by dark red and dark blue upward arrows, respectively. Polyadenylation sites and 3′-*trans*-splice sites mapped by end-reads in our RNA-Seq for all β-tubulin/α-tubulin regions are indicated by red and blue downward arrows, respectively. The numbers on top of the arrows designate the number of reads. Asterisk at the beginning of the sequence indicates the β-tubulin stop codon and asterisk at the end of the sequence indicates the α-tubulin start codon.
(0.07 MB PDF)

Figure S11 Experimental validation of multiple primary trans-splice sites for *T. brucei* genes. Overlay of the number of reads (log_2_) from 5′-end- (blue) and 3′-end-enriched (red) libraries aligning to the shown regions of chromosomes IV (A–C) and V (D) covering the ORFs for Tb927.4.1020 (A), Tb927.4.1180 (B), Tb927.4.1600 (C) and Tb927.5.990 (D). SL-containing end-reads are shown as blue or red horizontal lines depending on their orientation (minus or plus strand, respectively). Dashed lines indicate the positions of a nested gene-specific primer and the expected positions of the 3′ *trans*-splice sites. Green bars indicate the potential products from an RT-PCR assay with SL and gene-specific primers with the predicted size of the fragments indicated on the left. (E) RT-PCR assay. Poly(A)^+^ RNA was reverse transcribed with random primers and the resulting cDNA was used as a template for nested PCR with an identical SL forward primer for both amplification steps. Tb927.5.990 is an example of a gene with highly homogeneous site for SL addition.
(0.14 MB PDF)

Figure S12 Outline of the protocol for generation of 5′-triphosphate-end-enriched library for RNA-Seq. Generation and sequencing of a cDNA library enriched for 5′-triphosphate RNA ends, the hallmark of a 5′ end generated by an RNA polymerase.
(0.05 MB PDF)

Figure S13 Comparison between normalized (A) and non-normalized 5′-triphosphate-end-enriched library (B). Shown is a segment of chromosome VII surrounding a strand-switch region (SSR) of divergent transcription. Individual ORFs are indicated by bars colored based on their orientation. Grey arrows indicate the direction of transcription. The fold enrichment of reads (A) [(number of reads in the 5′-triphosphate-end-enriched library)×24/(number of reads in the 5′-end enriched library)] is plotted for the plus strand (red) and the minus strand (blue). The non-normalized number of reads (B) is shown for the plus strand (red) and the minus strand (blue).
(0.17 MB PDF)

Figure S14 Comparison between the mapped splice sites and polyadenylation sites in this study and the data set in [17]. For the splice sites (A) and poly(A) sites (B), we defined a per-gene measure of overlap between the data in this study and the data in Siegel et al. 2010, as follows: define the entire set of sites found by both studies as s_i_, for i = 1,…n. Define (in each study) the probability to observe the i'th site, p_i_, as the number of reads for that site divided by the total number of reads for the gene. The overlap is the sum over all sites of min(p_i_(this study), p_i_(Siegel et al 2010)). This gives 1, if there is perfect overlap and 0, if there is no overlap at all.
(0.06 MB PDF)

Figure S15 Abundance comparison between the data set in this study and that in [17]. Pairwise (gene-by-gene) comparison of RNA-Seq-based gene abundance reported previously with those derived from the current study (tabulated in Table S6). Correlation coefficients between the sets are 0.697 (Pearson) and 0.483 (Spearman).
(0.05 MB PDF)

Supporting Information S1 Additional description of materials and methods.
(0.17 MB PDF)

Table S1 List of predicted ORFs in GeneDB v_4 with a misannotated translation start codon, i.e. the SAS (the transcript 5′ end) mapped within the ORF.
(0.09 MB XLS)

Table S2 List of annotated ORFs in GeneDB v_4 not producing a detectable transcript in this analysis.
(0.11 MB XLS)

Table S3 List of genes that have an alternative processing site in the 5′ UTR or 3′ UTR.
(0.09 MB XLS)

Table S4 Listing of all mapped trans-splice acceptor sites (SAS) and measurement of the 5′UTR length. SAS mapping inside an ORF are indicated by NA in the 5′UTR length column. 5′UTRs were not defined for novel transcripts (NA).
(3.67 MB XLS)

Table S5 Listing of all mapped poly (A) addition sites (PAS) and measurement of the 3′UTR length. 3′UTRs were not defined for novel transcripts (NA).
(5.05 MB XLS)

Table S6 Abundance (RNAs/cell) of all transcripts detected in this analysis.
(1.32 MB XLS)

Table S7 List of all novel transcripts in *T. brucei* procyclic cells identified in this study.
(0.18 MB XLS)

Table S8 List of novel transcripts coding for ORFs with homology to annotated gene products in *T. cruzi* and/or *L. major*.
(0.05 MB XLS)

Table S9 List of novel transcripts coding for ORFs with homology to non-annotated ORFs in *T. cruzi* and/or *L. major*.
(0.03 MB XLS)

Table S10 List of novel transcripts in *T. brucei* procyclic cells identified in this study with matching MS/MS peptides by Panigrahi et al. [32].
(0.05 MB XLS)

Table S11 List of putative non-coding transcripts.
(0.02 MB XLS)

Table S12 Splice site (SAS) dispersion.
(0.86 MB XLS)

Table S13 Poly (A) site (PAS) dispersion.
(0.23 MB XLS)

Table S14 Putative Pol II transcription units in GeneDB v_4.
(0.04 MB XLS)
