# Supplementary material for: The Transcriptome of the Human Pathogen Trypanosoma brucei at Single-Nucleotide Resolution
Source: PLoS Pathog. 2010 Sep 9;6(9):e1001090. doi: 10.1371/journal.ppat.1001090 (PMC2936537; doi:10.1371/journal.ppat.1001090)
Supplement: Figure S1 — Comparison of technical and biological replica data sets. (A–I) Transcript abundance measures were derived from the number of reads (not including end-reads) that align within a 500-nucleotide window at the 5′ end of the transcript (for the SL-libraries) or at the 3′ end (for the poly-A enriched libraries; random primed or oligo(dT) primed). Shown above each graph are the Pearson's correlation coefficient (ρP) and the Spearman's rank correlation coefficient (ρS). Pcr, pct and pcs indicate libraries from procyclic cells prepared with random primers, oligo(dT) primer and SL primer (for the second cDNA strand), respectively. (0.64 MB PDF) [file ppat.1001090.s001.pdf]

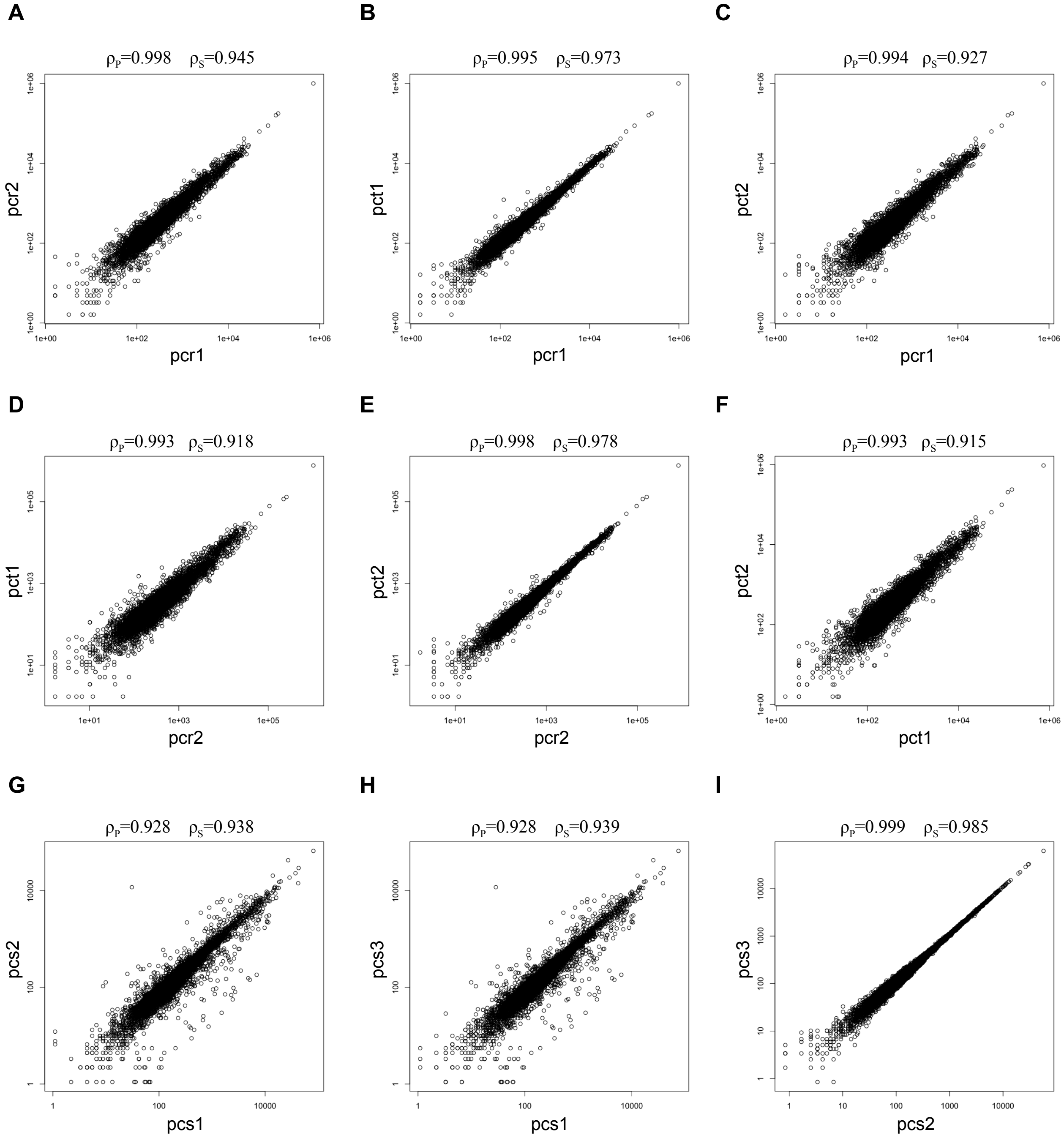

Figure S1. Comparison of technical and biological replica data sets. (*A-I*) Transcript abundance measures were derived from the number of reads (not including end-reads) that align within a 500-nucleotide window at the 5' end of the transcript (for the SL-libraries) or at the 3' end (for the poly-A enriched libraries; random primed or oligo(dT) primed). Shown above each graph are the Pearson's correlation coefficient (ρ_P) and the Spearman's rank correlation coefficient (ρ_S). *Pcr*, *pct* and *pcs* indicate libraries from procyclic cells prepared with random primers, oligo(dT) primer and SL primer (for the second cDNA strand), respectively.
