# Supplementary material for: The Transcriptome of the Human Pathogen Trypanosoma brucei at Single-Nucleotide Resolution
Source: PLoS Pathog. 2010 Sep 9;6(9):e1001090. doi: 10.1371/journal.ppat.1001090 (PMC2936537; doi:10.1371/journal.ppat.1001090)
Supplement: Figure S2 — Accuracy of alignment of sequence reads to the T. brucei reference genome. Shown is the overlay of the number of reads (log2) from 5′-end- (blue) and 3′-end-enriched (red) libraries aligning to ∼4kb window on chromosome III that contains the ORF for Tn10 transposase (Tb927.3.1050). Numbers of end-reads [SL-containing, blue; poly(A)-containing, red] are also shown (−log2). ORFs are represented by black arrows. No reads align to the Tn10 transposase ORF, which clearly is inserted within the sequence of a single transcript that covers both Tb927.3.1040 and Tb927.3.1060 (both currently annotated as interrupted, conserved hypothetical protein pseudogenes). According to GeneDB (www.genedb.org), the Tn10 insertion is an artifact from BAC construction and is not present in the Trypanosoma brucei brucei strain 927 genomic DNA or other BACs covering this region [1]. (0.07 MB PDF) [file ppat.1001090.s002.pdf]

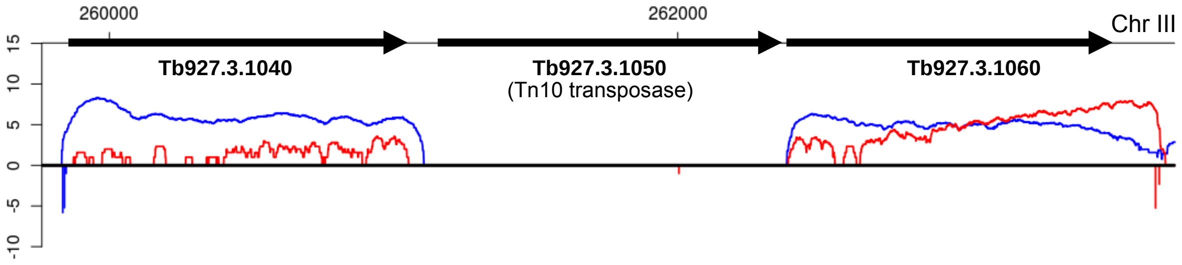

Figure S2. Accuracy of alignment of sequence reads to the *T. brucei* reference genome. Shown is the overlay of the number of reads (log_2) from 5′-end- (blue) and 3′-end-enriched (red) libraries aligning to ~4kb window on chromosome III that contains the ORF for Tn10 transposase (Tb927.3.1050). Numbers of end-reads [SL-containing, blue; poly(A)-containing, red] are also shown (-log_2). ORFs are represented by black arrows. No reads align to the Tn10 transposase ORF, which clearly is inserted within the sequence of a single transcript that covers both Tb927.3.1040 and Tb927.3.1060 (both currently annotated as interrupted, conserved hypothetical protein pseudogenes). According to GeneDB (www.genedb.org), the Tn10 insertion is an artifact from BAC construction and is not present in the *Trypanosoma brucei brucei* strain 927 genomic DNA or other BACs covering this region [1].
