# Supplementary material for: The Transcriptome of the Human Pathogen Trypanosoma brucei at Single-Nucleotide Resolution
Source: PLoS Pathog. 2010 Sep 9;6(9):e1001090. doi: 10.1371/journal.ppat.1001090 (PMC2936537; doi:10.1371/journal.ppat.1001090)
Supplement: Figure S3 — Experimental validation of misannotated translation start codons in T. brucei genes. Overlay of the number of reads (log2) from 5′-end- (blue) and 3′-end- (red) enriched libraries aligning to the shown regions of chromosome VIII covering the ORFs for Tb927.8.1270 (A) and Tb927.8.2000 (B). Numbers of end-reads (−log2) are also shown [SL, blue; poly(A), red]. Dashed lines indicate the positions of a gene-specific primer, the newly annotated trans-splice site and the currently annotated ATG for each of the two genes. Green bars indicate the potential products from an RT-PCR assay with SL and gene-specific primers. (C) RT-PCR assay. Poly(A)+ RNA was reverse transcribed with random primers and the resulting cDNA was used as a template for nested PCR with an identical SL forward primer for both amplification steps. Nested PCR was used to ensure specificity of amplification since the forward SL primer anneals to cDNA products from all T. brucei mRNAs. The sizes of the amplified products indicate that the ORFs for the corresponding genes are shorter than currently annotated. (0.13 MB PDF) [file ppat.1001090.s003.pdf]

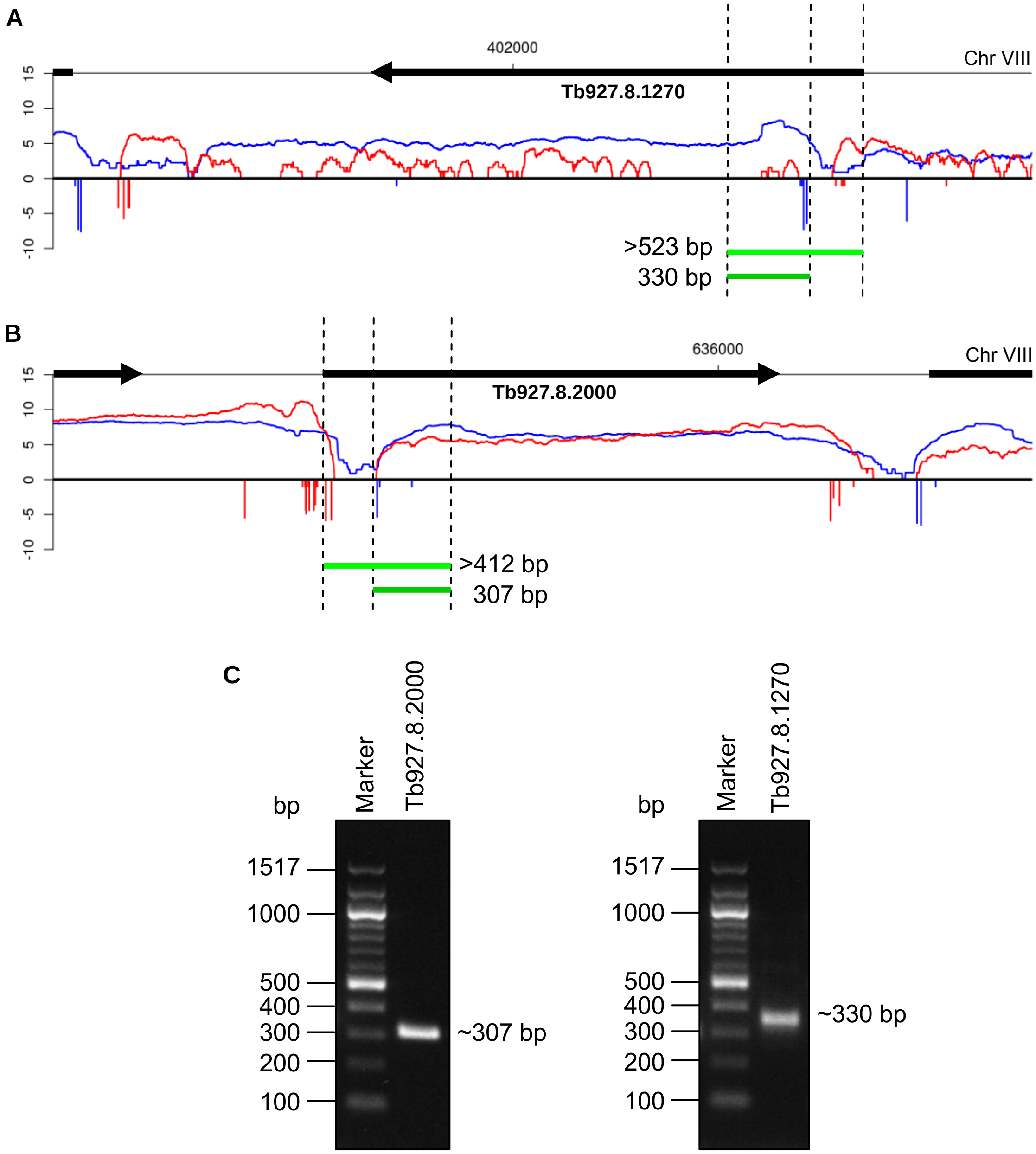

Figure S3. Experimental validation of misannotated translation start codons in *T. brucei* genes. Overlay of the number of reads (log_2) from 5′-end- (blue) and 3′-end- (red) enriched libraries aligning to the shown regions of chromosome VIII covering the ORFs for Tb927.8.1270 (*A*) and Tb927.8.2000 (*B*). Numbers of end-reads ($-log_2$) are also shown [SL, blue; poly(A), red]. Dashed lines indicate the positions of a gene-specific primer, the newly annotated *trans*-splice site and the currently annotated ATG for each of the two genes. Green bars indicate the potential products from an RT-PCR assay with SL and gene-specific primers. (*C*) RT-PCR assay. Poly(A)+ RNA was reverse transcribed with random primers and the resulting cDNA was used as a template for nested PCR with an identical SL forward primer for both amplification steps. Nested PCR was used to ensure specificity of amplification since the forward SL primer anneals to cDNA products from all *T. brucei* mRNAs. The sizes of the amplified products indicate that the ORFs for the corresponding genes are shorter than currently annotated.
