# Supplementary material for: The Transcriptome of the Human Pathogen Trypanosoma brucei at Single-Nucleotide Resolution
Source: PLoS Pathog. 2010 Sep 9;6(9):e1001090. doi: 10.1371/journal.ppat.1001090 (PMC2936537; doi:10.1371/journal.ppat.1001090)
Supplement: Figure S4 — Experimental validation of misannotated T. brucei genes that are part of transcripts from neighboring genes. Overlay of the number of reads (log2) from 5′-end- (blue) and 3′-end-enriched (red) libraries aligning to the shown regions of chromosomes VIII (A) and I (B). Black arrows represent ORFs encoding conserved proteins and grey arrows represent ORFs for a sequence orphan Tb927.8.5790 (A) and the unlikely hypothetical proteins Tb927.1.3060, Tb927.1.3080, Tb927.1.3090 and Tb927.1.3100 (B). (C) Northern blots of total RNA fractionated on denaturing agarose gels with probes against the indicated ORFs (adjacent lanes of the gel were used for the blots with different probes). The probes against Tb927.8.5780 and Tb927.8.5790 are detecting the same size transcript of ∼2.9 kb [∼2.8 kb plus a poly(A) tail]. The probes against Tb927.1.3070 and Tb927.1.3100 are also detecting the same size transcript of ∼2.6 kb [∼2.5 kb plus a poly(A) tail]. Positions of markers are indicated on the left. (0.13 MB PDF) [file ppat.1001090.s004.pdf]

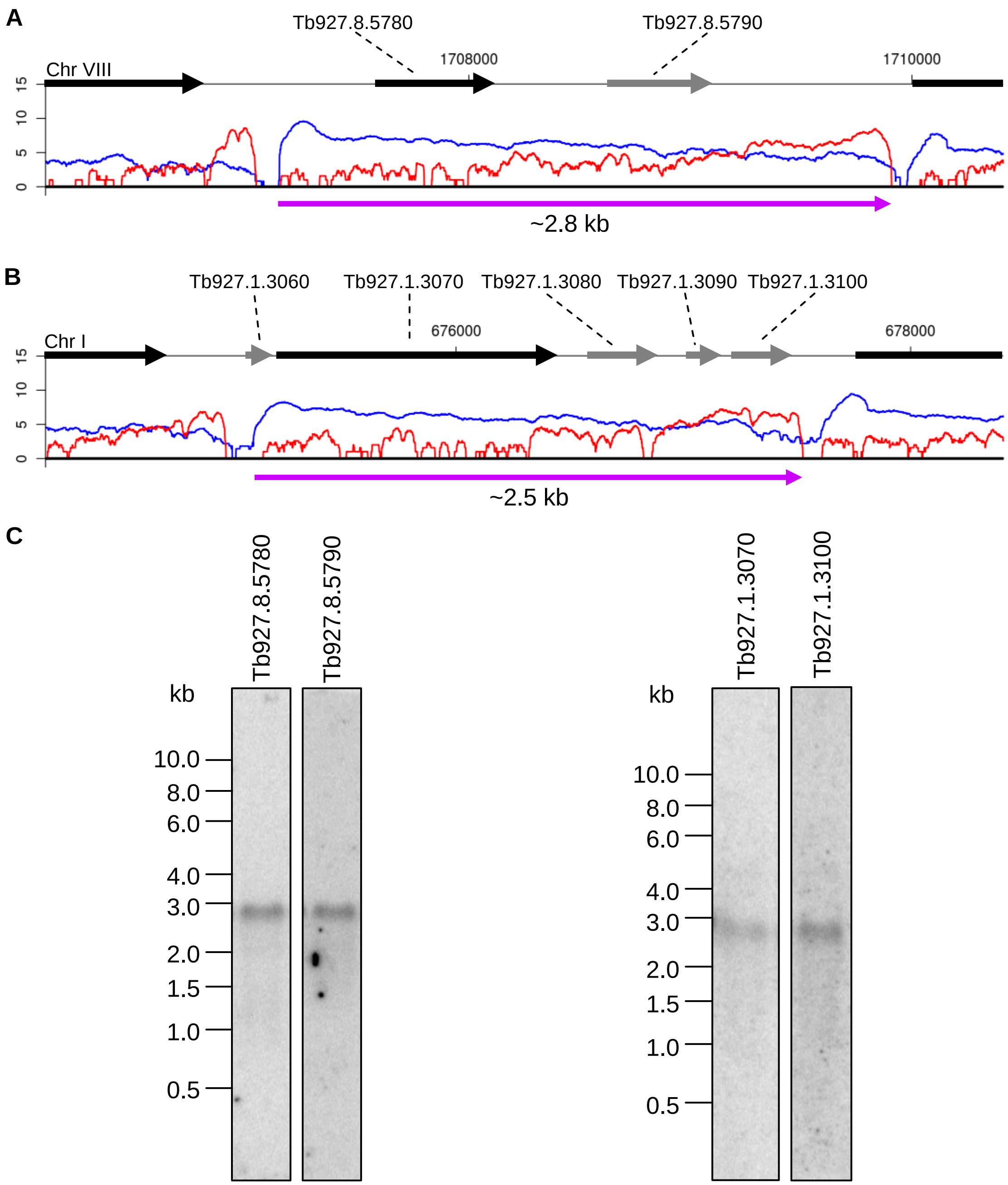

Figure S4. Experimental validation of misannotated *T. brucei* genes that are part of transcripts from neighboring genes. Overlay of the number of reads ($\log_2$) from 5′-end- (blue) and 3′-end-enriched (red) libraries aligning to the shown regions of chromosomes VIII (*A*) and I (*B*). Black arrows represent ORFs encoding conserved proteins and grey arrows represent ORFs for a sequence orphan Tb927.8.5790 (*A*) and the unlikely hypothetical proteins Tb927.1.3060, Tb927.1.3080, Tb927.1.3090 and Tb927.1.3100 (*B*). (*C*) Northern blots of total RNA fractionated on denaturing agarose gels with probes against the indicated ORFs (adjacent lanes of the gel were used for the blots with different probes). The probes against Tb927.8.5780 and Tb927.8.5790 are detecting the same size transcript of ~2.9 kb [~2.8 kb plus a poly(A) tail]. The probes against Tb927.1.3070 and Tb927.1.3100 are also detecting the same size transcript of ~2.6 kb [~2.5 kb plus a poly(A) tail]. Positions of markers are indicated on the left.
