# Supplementary material for: The Transcriptome of the Human Pathogen Trypanosoma brucei at Single-Nucleotide Resolution
Source: PLoS Pathog. 2010 Sep 9;6(9):e1001090. doi: 10.1371/journal.ppat.1001090 (PMC2936537; doi:10.1371/journal.ppat.1001090)
Supplement: Figure S5 — Experimental validation of T. brucei genes that produce alternatively processed transcripts. Overlay of the number of reads (log2) from 5′-end- (blue) and 3′-end-enriched (red) libraries aligning to the shown regions of chromosome IV covering the transcripts produced for Tb927.4.4370 (A) and Tb927.4.4490 (B). Black arrows represent the annotated ORFs and purple arrows represent transcripts suggested by the internal tags (peaks in the pileups are indicated with red and blue downward arrows) and end-reads (not shown) alignment to the T. brucei genome. Note that Tb927.4.4370 is another example of a gene with a misanotated translation start codon. The positions of the regions hybridizing to specific probes are indicated by short black lines. (C) Northern blots of total RNA fractionated on denaturing agarose gels with the indicated (a–d) probes. Both probes a and b detect the full-length Tb927.4.4370 transcript. Probe a additionally detects a shorter transcript containing the Tb927.4.4370 ORF, while probe b additionally detects a shorter transcript that is a part of the 3′ UTR of the full-length Tb927.4.4370 transcript. Probes c and d both detect the full-length Tb927.4.4490 transcript. Probe c additionally detects a shorter transcript containing the Tb927.4.4490 ORF, while probe d additionally detects a shorter transcript that is a part of the 3′ UTR of the full-length Tb927.4.4490 transcript. Positions of marker RNA bands are indicated on the left. (0.17 MB PDF) [file ppat.1001090.s005.pdf]

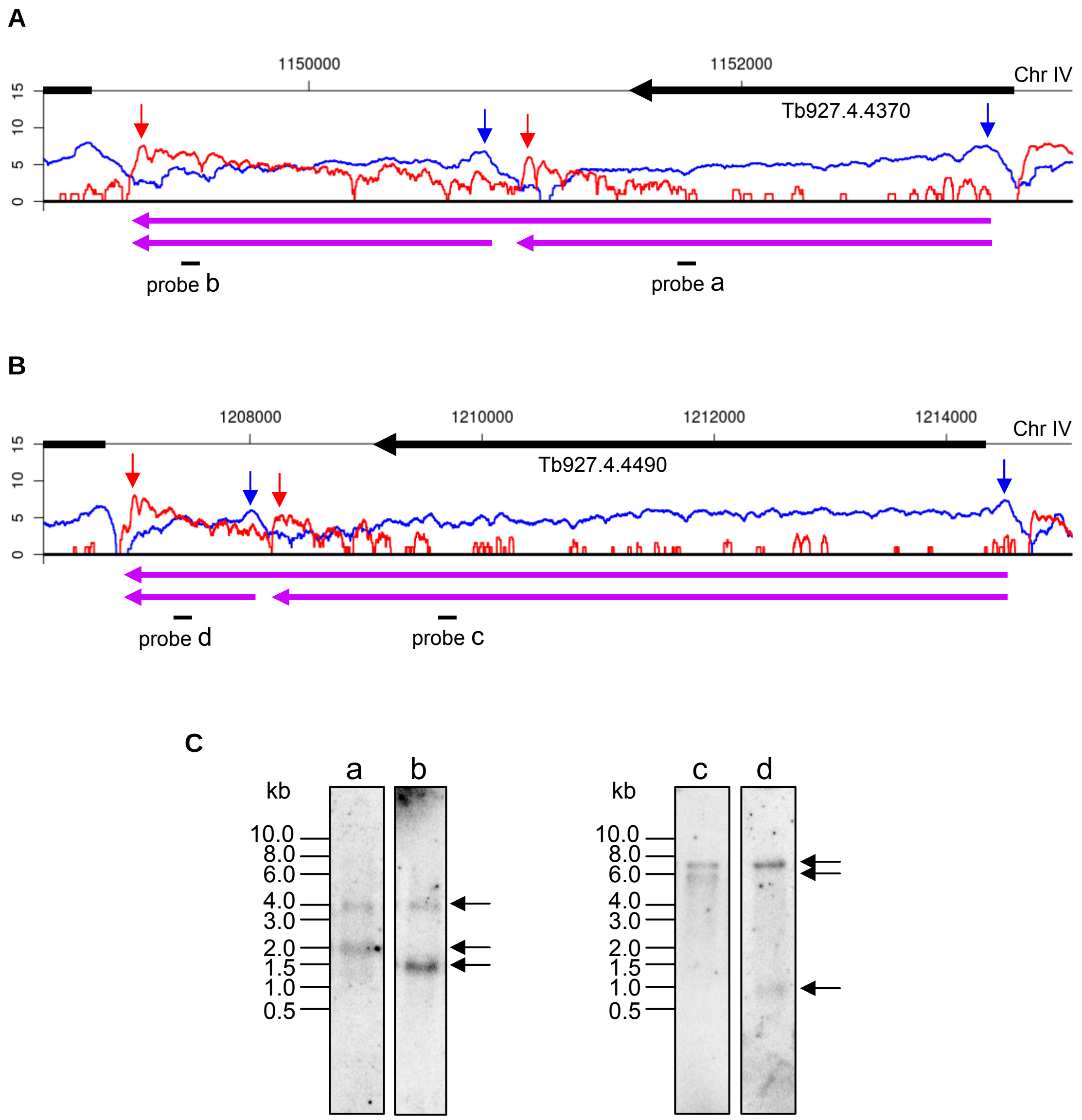

Figure S5. Experimental validation of *T. brucei* genes that produce alternatively processed transcripts. Overlay of the number of reads ($\log_2$) from 5′-end- (blue) and 3′-end-enriched (red) libraries aligning to the shown regions of chromosome IV covering the transcripts produced for Tb927.4.4370 (*A*) and Tb927.4.4490 (*B*). Black arrows represent the annotated ORFs and purple arrows represent transcripts suggested by the internal tags (peaks in the pileups are indicated with red and blue downward arrows) and end-reads (not shown) alignment to the *T. brucei* genome. Note that Tb927.4.4370 is another example of a gene with a misanotated translation start codon. The positions of the regions hybridizing to specific probes are indicated by short black lines. (*C*) Northern blots of total RNA fractionated on denaturing agarose gels with the indicated (a – d) probes. Both probes a and b detect the full-length Tb927.4.4370 transcript. Probe a additionally detects a shorter transcript containing the Tb927.4.4370 ORF, while probe b additionally detects a shorter transcript that is a part of the 3′ UTR of the full-length Tb927.4.4370 transcript. Probes c and d both detect the full-length Tb927.4.4490 transcript. Probe c additionally detects a shorter transcript containing the Tb927.4.4490 ORF, while probe d additionally detects a shorter transcript that is a part of the 3′ UTR of the full-length Tb927.4.4490 transcript. Positions of marker RNA bands are indicated on the left.
