# Supplementary material for: The Transcriptome of the Human Pathogen Trypanosoma brucei at Single-Nucleotide Resolution
Source: PLoS Pathog. 2010 Sep 9;6(9):e1001090. doi: 10.1371/journal.ppat.1001090 (PMC2936537; doi:10.1371/journal.ppat.1001090)
Supplement: Figure S6 — A novel transcript with limited coding potential is possibly associated with polyribosomes. (A) Cell extracts prepared in the presence of pactamycin (an antibiotic that promotes apparent polyribosome dissociation through inhibition of the formation of complete translation initiation complexes) or cycloheximide (an antibiotic that arrests polyribosomes by interfering with ribosome translocation) were subjected to sucrose gradient ultracentrifugation. RNA was purified from individual gradient fractions, separated on a denaturing agarose gel, transferred and cross-linked to a nylon membrane, and large rRNAs were visualized by staining with methylene blue. Shown is every other fraction from the gradient. (B) Northern blot of the fractionated RNA with a probe detecting the full-length Tb927.4.4370 transcript (FL) and a shorter transcript that is a part of the 3′UTR of the full-length Tb927.4.4370 transcript (3′UTR) as a result of alternative processing (probe b in Supplemental Fig. S5). (C) Northern blot with a probe detecting a transcript from a novel gene on chromosome X (Tb10.NT.122). (D) Northern blot with a probe against α-tubulin transcripts. Note the shift of α-tubulin mRNA from polysome fractions of the gradient in the cycloheximide-treated extract to lighter gradient fractions in the pactamycin-treated extract. A similar shift is seen for Tb10.NT.122 (C). Three frame translation for Tb927.4.4370 3′UTR transcript (E) and Tb10.NT.122 (F) highlighting the limited coding potential of these transcripts. All ORFs are colored orange. (0.20 MB PDF) [file ppat.1001090.s006.pdf]

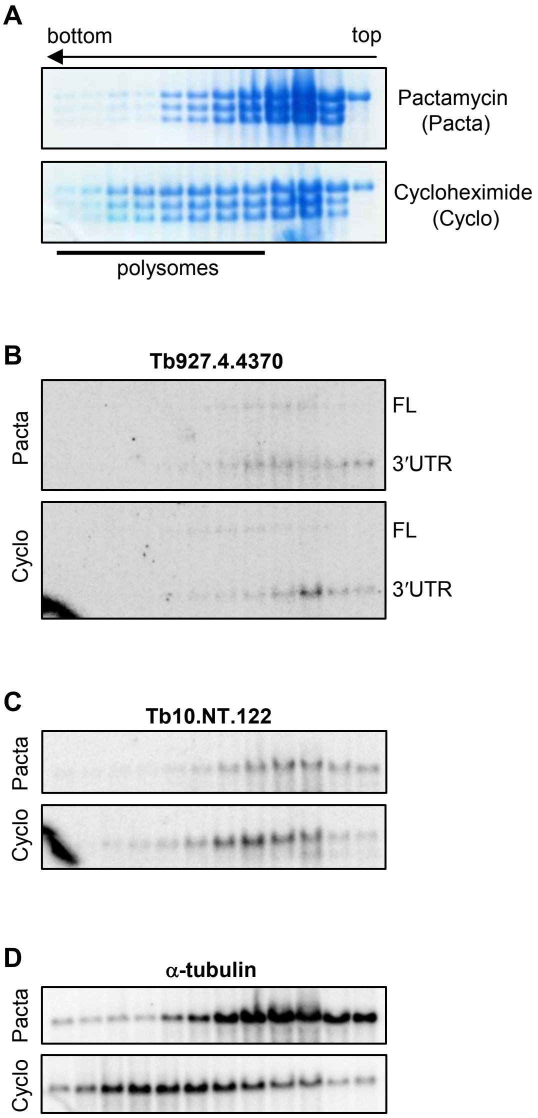

E

Tb927.4.4370 3′UTR transcript translation

Frame 1
Met P S E G K Met R G T H L R K K K Met R K Stop S R A G R E E Q S Stop N I Stop Stop A K E G
R G T A Stop F F P E K R N S V R Y L S S Stop H G L V A V F F Y C Y P L K H T C V Y I Y I Y V A S
F A H G K H R L L Stop C G L E P K Q L V P F K T G R N F F E Y C E G V R N V P F L G D A E T Q L
V G E R N C W S F Met L A C A Stop Q A T L I V K E E R G Stop K Stop D A N P Q N R T H F N F V I
L E R K C L N F S S K A C G E A T A E Met C K C R W N V L R N Q Met V I L A L H G C C C C C R F
V L F C T T L Stop I A R R G G G R L K V Stop V V Q T H L Stop V K G G K C K L L Y N G A R I Y N
R N F N K A R Met Stop R G V T A D T Q R K Q L Q K Q H Y N Y G F D T L R P S P R T H H Q Y C L
V V S S S F H V F V V R P E G S V L V N G H I D S T Stop K K Q N F V S G C R V T S H V T T N E V
E V T G D F I W T T E E T T S W W L P E W I L S S Q S N E K H D R L W L C I N S N G V K K S S S S
Stop H C G R R S G Met R W S L C Q T H L T A F L S I S I K E Q V Stop K K K K K E G F L T C G
Stop L F V L L Met L L L Met L L S C V C V L E R Y L A I H Q L I R W F L I V H C I C L Met V T Y V V
S I Stop Q H T S P V Stop T G S D S K N Q K G Stop L Q Met K D G A C V R V F T F S F T F Y F F P
P F P V N A V Met G S V A P Y G S I R Y E F Y Q

Frame 2
C L P R V K Stop E E R I Y E K K K C V S R V G Q G A R N S R E I S N E Q K K E G G L R D F F R K
K E I A F G I S Q V S Met A S L Q Y F F I V T H Stop S T P A Y I Y I Y Met L H H L H Met V N T G C S
D A G Stop S L S N Stop S P S K L V G T F L N T V R V C E T F H F Stop A Met L R R S Stop S V N
V I V G P S C L L A R D R R R Stop L Stop R R K G G K N K T Q I R R T V L T L T L Stop Y Stop R E
N A Stop I L V V K L A E R P R R R C A S V A G T S Stop G I K W L S Stop R C Met V A V A V V V L
S C F V P P Y E S P A A E A A D S K C K L C R H T C K Stop K G E N V N C C T Met E P A Y I T A T
S T R H G C D V A Stop Q Q I H N E S N F R S S I I I Met G S I R Stop D L L H G H I I S I V L S F H L
H F T S L S C V Q K D Q F L Stop Met D I L T V R E R N K I L C R V V V L R H T L Q P T K Stop K S
P E T S Y G P P K K R H R G G S R S G Y Stop A V K A Met R N Met T A F G C A L I Q T V Stop K R
V P V R D I V V V E V A C G G P S V R P T S L R F F Q Y Q L K S R C K R K R K R R V F Stop R V G
D Y S F C Stop C C C Stop C C C L V C A C Stop S D I L R Y T N Stop F D G F Stop L C T V S V
Stop W L P Met L Stop A F D N T L H L S R R D L I P K I R R D S Y K Stop R T G R A S G F L P F H
S P F I F F H H F P Stop Met L L W V L S H L Met G V Y D T S S I

Frame 3
A F R G Stop N E R N A F T K K K N A Stop V E Stop G R A R G T V V K Y L Met S K R R K G D C V
I F S G K K K Stop R S V S L K L A W P R C S I F L L L P T E A H L R I Y I Y I C C I I C T W Stop T Q
V A L Met R V R A Stop A T S P L Q N W S E L F Stop I L Stop G C A K R S I F R R C Stop D A V S
R Stop T Stop L L V L H A C L R V T G D V D C E G G K G V K I R R K S A E P Y S L Stop L C D I R
E K Met L E F Stop Stop Stop S L R R G H G G D V Q V S L E R P E E S N G Y P S A A W L L L L L
S F C L V L Y H L Met N R P P R R R P T Q S V S C A D T P V S E R G K Met Stop I A V Q W S P H I
Stop P Q L Q Q G T D V T W R D S R Y T T K A T S E A A L Stop L W V R Y A E T F S T D T S S V L
S C R F I F I S R L C R A S R R I S S C E W T Y Stop Q Y V K E T K F C V G L S C Y V T R Y N Q R S
R S H R R L H Met D H R R N D I V V A P G V D I K Q S K Q Stop E T Stop P P L V V H Stop F K R C
K K E F Q F V T L W S S K W H A V V P L S D P P H C V S F N I N Stop R A G V K E K E K G G F F N
V W V T I R F V D V V V D V V V L C V R V R A I S C D T P I D S Met V F D C A L Y L F N G Y L C C E
H L T T H F T C L D G I Stop F Q K S E G I A T N E G R G V R P G F Y L F I H L L F F S T I S R K C C
Y G F C R T L W E Y T I R V L S

F

Tb10.NT.122 translation

Frame 1
H S S T T S K G G K S C E H N N F S Y Y V T V V L A L D T S H K Stop S G V T Stop A V T D R F T P
Stop L I T S L H I I A V V I F R K L F Met R P F L F L N N L F G Met L F W E Y Stop C L L F G F R Y E
D L I P I Met T Y Stop Y R N S T Q Y N C F Q F T G N E G R G K H R G I L V V Stop T R N N C E V R
V Y C L T L T L L A L F K D E Y D D P V Stop R P V G R V F D V I T S Stop G Y I F G L N N I E V P C
Stop Y L Y G N L G I F Stop K A T V P Q Y T Q V G S V H P L L F I F E S W V C T Stop W P Y C T R I
Q C I G T L I T R Stop V D D R S W Met H S F Stop R Met A R L T C K Stop L L Stop R T T S Y E I R
Y V Y V V I S F L R Q L Y T R I I Y G H P C G S F R C T I F Stop T R V C E E A S T L E S K R R S S T
F V Met L S D W K L T R S S K Met Met A L G D R A R T F I G Stop A T P T F E Stop H I Q L R V T H
Met Stop Y Q L W K K R H E K A V F I I C W E K V T

Frame 2
I Q V Q H L K V E K V V N T I I L A I T L P Stop Y W R W I R R T S N R G S L E L L L T D L H R N
Stop Stop R R F I S L L L L F F E N Y L C D L F C S L T I C S A C C F G S T N A Y C L D F V Met K I
L S Q Stop Stop H T N I G T A R S T I A F N S L A T K G G E N T G E Y W W F K H A I I V K C V F I A
L H L R Y Stop H S S K Met S T Met T L C S A P S G V S L Met Stop Stop R H R G I Y L D Stop T I
Stop K C R A D T F T V T Stop V F S R R L L F L S I L R Stop A L F I P Y Y S Y S S L G S A L S G L I
V Q G Y N V L V P L L Q G K L T T E V G C T V F D A W Q G Stop H V S N Y Y E E R R L T K Stop D
T F Met W Stop F L S C A N C T L V L Y Met V T H A V V S G V R Y F R R G S V K K P Q R Stop S Q
R E G A V L S Stop C C L T G N L L V A A K Stop W H S A T E R A H S Stop G K Q P R R S S D I Y S
Stop E L L T C D I N Y G K N G Met K K Q Y S L F V G K K L P

Frame 3
F K Y N I Stop R W K K L Stop T Q Stop F Stop L L R Y R S I G V G Y V A Q V I G G H L S C Y Stop
Q I Y T V T D N V A S Y H C C C Y F S K T I Y A T F S V P Stop Q F V R H A V L G V L Met L I V W I S
L Stop R S Y P N N D I L I Stop E Q H A V Q L L S I H W Q R R A G K T P G N T G G L N T Q Stop L
Stop S A C L L P Y T Y V I S T L Q R Stop V R Stop P C V A P R R A C L Stop C D N V I G V Y I W T
E Q Y R S A V L I P L R Stop P R Y F L E G Y C S S V Y S G R L C S S L I I H I R V L G L H L V A L L
Y K D T Met Y W Y P Y Y K V S Stop R Q K L D A Q F L T H G K A D Met Stop V I T Met K N D V L R
N K I R L C G N F F L A P T V H S Y Y I W S P Met R Stop F Q V Y D I L D E G L Stop R S L N V R V
K E K E Q Y F R N V V Stop L E T Y S Stop Q Q N D G T R R Q S A H I H R V S N P D V R V T Y T V
K S Y S H V I S I Met E K T A Stop K S S I H Y L L G K S Y Q

Figure S6. A novel transcript with limited coding potential is possibly associated with polyribosomes. (*A*) Cell extracts prepared in the presence of pactamycin (an antibiotic that promotes apparent polyribosome dissociation through inhibition of the formation of complete translation initiation complexes) or cycloheximide (an antibiotic that arrests polyribosomes by interfering with ribosome translocation) were subjected to sucrose gradient ultracentrifugation. RNA was purified from individual gradient fractions, separated on a denaturing agarose gel, transferred and cross-linked to a nylon membrane, and large rRNAs were visualized by staining with methylene blue. Shown is every other fraction from the gradient. (*B*) Northern blot of the fractionated RNA with a probe detecting the full-length Tb927.4.4370 transcript (FL) and a shorter transcript that is a part of the 3′ UTR of the full-length Tb927.4.4370 transcript (3′ UTR) as a result of alternative processing (probe b in Supplemental Fig. S5). (*C*) Northern blot with a probe detecting a transcript from a novel gene on chromosome X (Tb10.NT.122). (*D*) Northern blot with a probe against α-tubulin transcripts. Note the shift of α-tubulin mRNA from polysome fractions of the gradient in the cycloheximide-treated extract to lighter gradient fractions in the pactamycin-treated extract. A similar shift is seen for Tb10.NT.122 (*C*). Three frame translation for Tb927.4.4370 3′ UTR transcript (*E*) and Tb10.NT.122 (*F*) highlighting the limited coding potential of these transcripts. All ORFs are colored orange.
