# Supplementary material for: The Transcriptome of the Human Pathogen Trypanosoma brucei at Single-Nucleotide Resolution
Source: PLoS Pathog. 2010 Sep 9;6(9):e1001090. doi: 10.1371/journal.ppat.1001090 (PMC2936537; doi:10.1371/journal.ppat.1001090)
Supplement: Figure S7 — Examples of snoRNA-precursor transcripts. (A) A transcript containing a single snoRNA. (B) Multiple snoRNAs embedded in the 3′ UTR of a protein coding transcript. (C) Multiple snoRNAs embedded in the ORF of a protein coding transcript. (D) A snoRNA cluster producing multiple precursor transcripts containing more than one snoRNA. All panels show the overlay of the number of reads (log2) from 5′-end- (blue) and 3′-end-enriched (red) libraries. Numbers of end-reads (−log2) are also shown (SL, blue; poly(A), red). Black arrows represent currently annotated ORFs and red arrowheads represent mature snoRNA sequences. (0.17 MB PDF) [file ppat.1001090.s007.pdf]

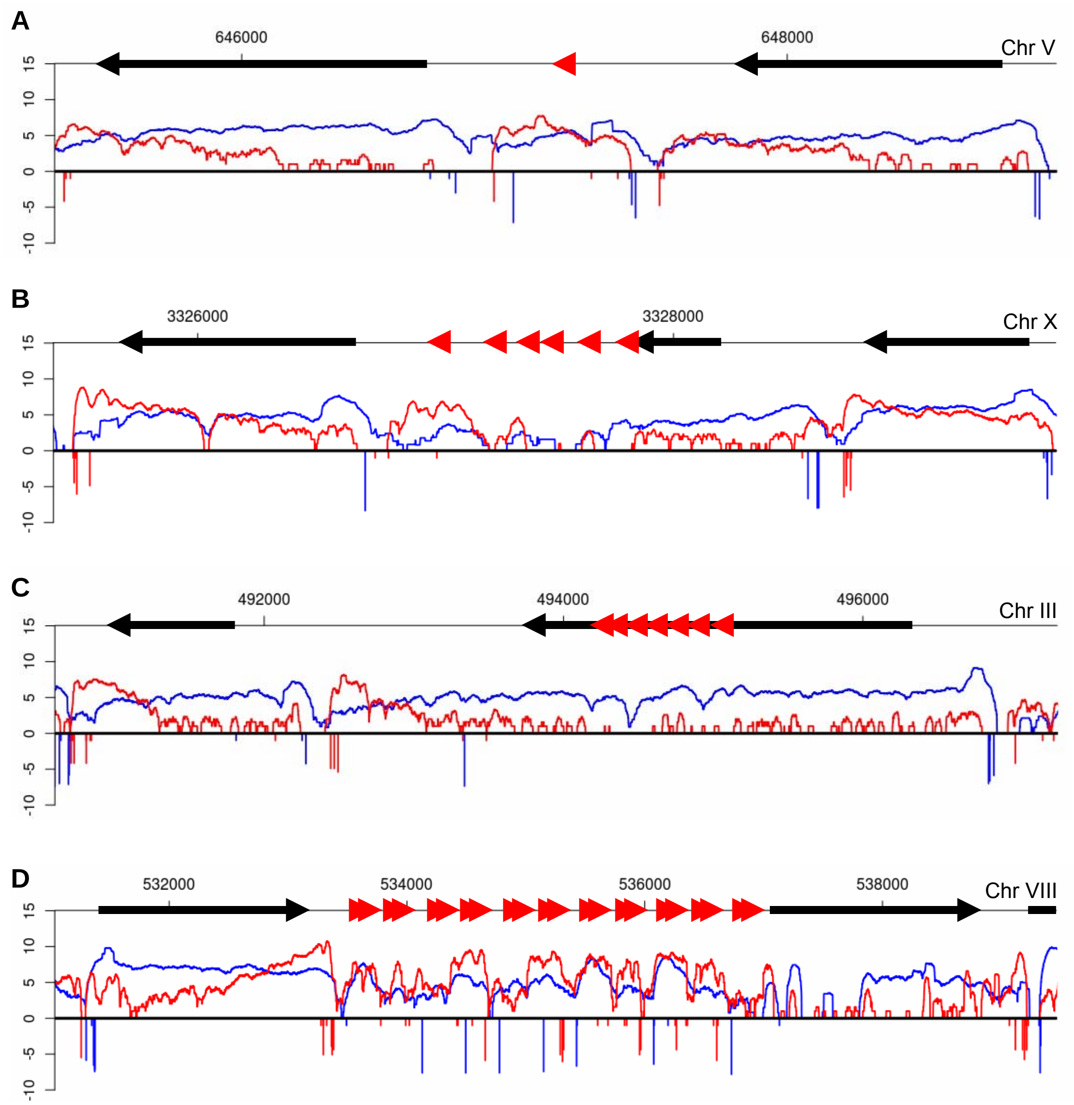

Figure S7. Examples of snoRNA-precursor transcripts. (*A*) A transcript containing a single snoRNA. (*B*) Multiple snoRNAs embedded in the 3′ UTR of a protein coding transcript. (*C*) Multiple snoRNAs embedded in the ORF of a protein coding transcript. (*D*) A snoRNA cluster producing multiple precursor transcripts containing more than one snoRNA. All panels show the overlay of the number of reads (log_2) from 5′-end- (blue) and 3′-end-enriched (red) libraries. Numbers of end-reads ($-log_2$) are also shown (SL, blue; poly(A), red). Black arrows represent currently annotated ORFs and red arrowheads represent mature snoRNA sequences.
