# Supplementary material for: The Transcriptome of the Human Pathogen Trypanosoma brucei at Single-Nucleotide Resolution
Source: PLoS Pathog. 2010 Sep 9;6(9):e1001090. doi: 10.1371/journal.ppat.1001090 (PMC2936537; doi:10.1371/journal.ppat.1001090)
Supplement: Figure S8 — Abundance profile of T. brucei transcripts. Relative transcript abundance represents the number of reads (not including end-reads) that align within a 500-nucleotide window at the 5′ end of the transcript (combined for the SL-library replicas) for each gene. Plotted is the number of genes with identical number of reads aligning to the 500 nt window. Genes without reads aligning to their sequence are not shown. Calculation of the estimated number of mRNA molecules per cell was based on using the precisely measured PGKB mRNA level in cultured procyclic T. brucei cells [28] as a reference point. Our estimates for mRNAs copy numbers in T. brucei closely resemble data obtained for yeast [29], [30] and mammals [31]. Darker shades of red background indicate higher copy numbers per cell. (0.04 MB PDF) [file ppat.1001090.s008.pdf]

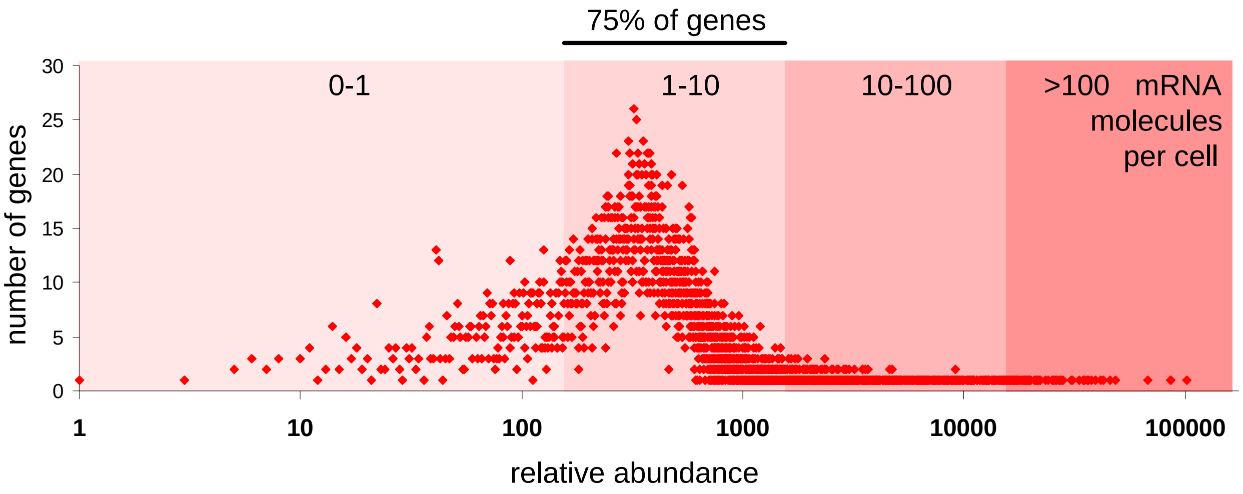


**Figure S8. Abundance profile of *T. brucei* transcripts.** Relative transcript abundance represents the number of reads (not including end-reads) that align within a 500-nucleotide window at the 5′ end of the transcript (combined for the SL-library replicas) for each gene. Plotted is the number of genes with identical number of reads aligning to the 500 nt window. Genes without reads aligning to their sequence are not shown. Calculation of the estimated number of mRNA molecules per cell was based on using the precisely measured PGKB mRNA level in cultured procyclic *T. brucei* cells [28] as a reference point. Our estimates for mRNAs copy numbers in *T. brucei* closely resemble data obtained for yeast [29,30] and mammals [31]. Darker shades of red background indicate higher copy numbers per cell.
