# Supplementary material for: The Transcriptome of the Human Pathogen Trypanosoma brucei at Single-Nucleotide Resolution
Source: PLoS Pathog. 2010 Sep 9;6(9):e1001090. doi: 10.1371/journal.ppat.1001090 (PMC2936537; doi:10.1371/journal.ppat.1001090)
Supplement: Figure S9 — Experimental validation of transcripts for novel T. brucei genes. (A) Overlay of the number of reads (log2) from 5′-end- (blue) and 3′-end-enriched (red) libraries aligning to the shown regions of chromosomes VIII and XI. Transcripts for novel genes (purple bars) are labeled a through e and their approximate sizes indicated [excluding the poly(A) tail]. (B) Sequence of the polypeptides encoded by putative ORFs in the indicated (a–e) transcripts. a, b, and c encode the T. brucei ribosomal protein L41. Also shown are the sequences for the three Leishmania braziliensis L41 polypeptides (encoded by three unannotated, tandemly arranged genes) and the human L41 sequence. Identical amino acids between the sequences from the three species are colored red. d and e encode 56 AA and 62 AA proteins respectively that are conserved but not annotated in L. braziliensis. (C) Northern blots of total RNA fractionated on denaturing agarose gels with probes against the indicated (a–e) transcripts. The (a, b, c) blot was performed with a probe against the short ORF present in all three transcripts. Transcripts a and b have almost identical size and they co-migrate during electrophoretic separation of the RNA sample. Positions of marker RNA bands are indicated on the left. (0.17 MB PDF) [file ppat.1001090.s009.pdf]

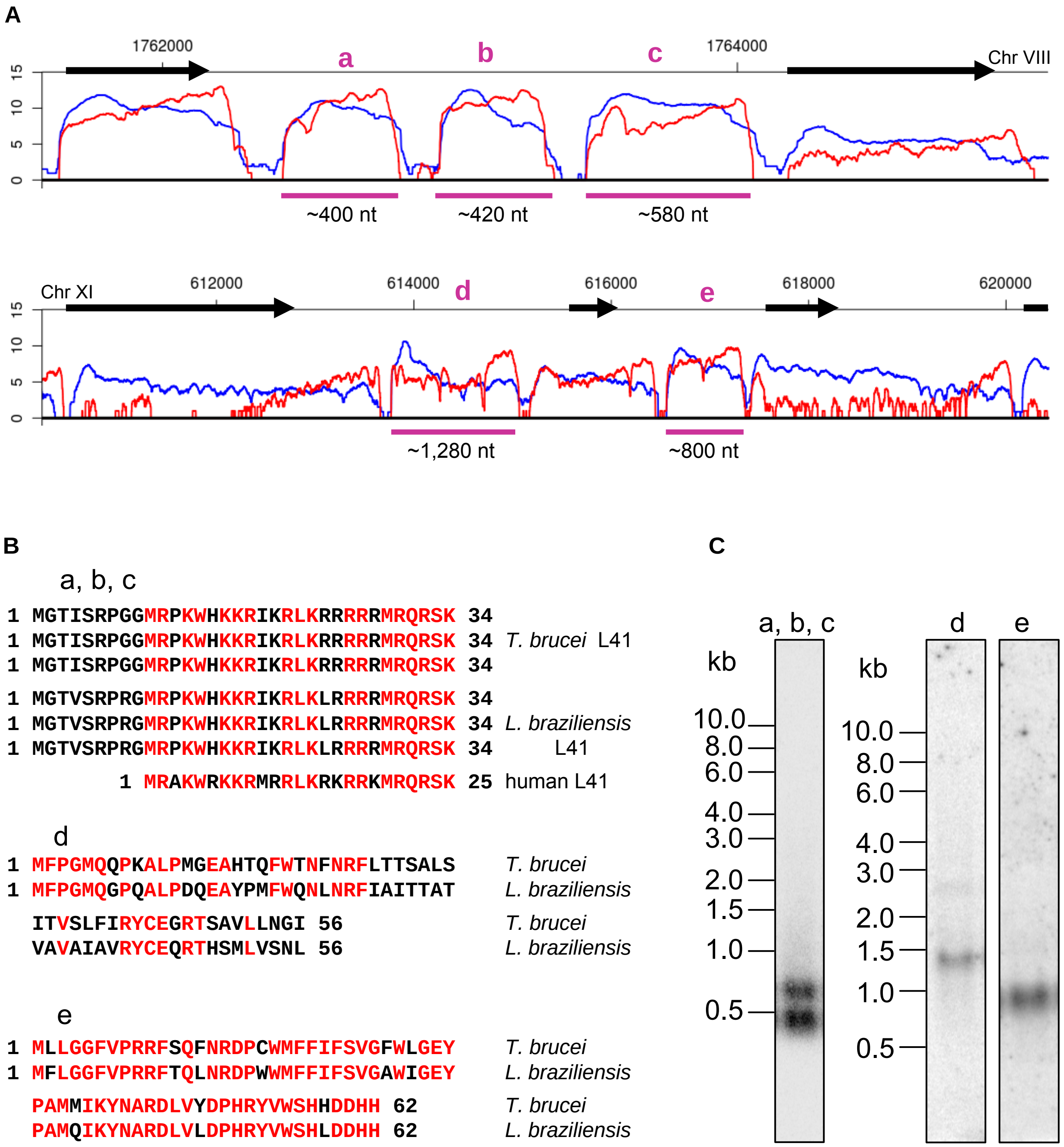

Figure S9. Experimental validation of transcripts for novel *T. brucei* genes. (*A*) Overlay of the number of reads ($\log_2$) from 5′-end- (blue) and 3′-end-enriched (red) libraries aligning to the shown regions of chromosomes VIII and XI. Transcripts for novel genes (purple bars) are labeled a through e and their approximate sizes indicated [excluding the poly(A) tail]. (*B*) Sequence of the polypeptides encoded by putative ORFs in the indicated (a – e) transcripts. a, b, and c encode the *T. brucei* ribosomal protein L41. Also shown are the sequences for the three *Leishmania braziliensis* L41 polypeptides (encoded by three unannotated, tandemly arranged genes) and the human L41 sequence. Identical amino acids between the sequences from the three species are colored red. d and e encode 56 AA and 62 AA proteins respectively that are conserved but not annotated in *L. braziliensis*. (*C*) Northern blots of total RNA fractionated on denaturing agarose gels with probes against the indicated (a – e) transcripts. The (a, b, c) blot was performed with a probe against the short ORF present in all three transcripts. Transcripts a and b have almost identical size and they co-migrate during electrophoretic separation of the RNA sample. Positions of marker RNA bands are indicated on the left.
