# Supplementary material for: The Transcriptome of the Human Pathogen Trypanosoma brucei at Single-Nucleotide Resolution
Source: PLoS Pathog. 2010 Sep 9;6(9):e1001090. doi: 10.1371/journal.ppat.1001090 (PMC2936537; doi:10.1371/journal.ppat.1001090)
Supplement: Figure S10 — Comparison between RNA-Seq mapped processing sites with sites mapped by previous sequencing of cDNA clones [8]. Polyadenylation sites and 3′-trans-splice sites mapped by cDNA clones sequencing in the β-tubulin/α-tubulin inter-ORF region are indicated by dark red and dark blue upward arrows, respectively. Polyadenylation sites and 3′-trans-splice sites mapped by end-reads in our RNA-Seq for all β-tubulin/α-tubulin regions are indicated by red and blue downward arrows, respectively. The numbers on top of the arrows designate the number of reads. Asterisk at the beginning of the sequence indicates the β-tubulin stop codon and asterisk at the end of the sequence indicates the α-tubulin start codon. (0.07 MB PDF) [file ppat.1001090.s010.pdf]

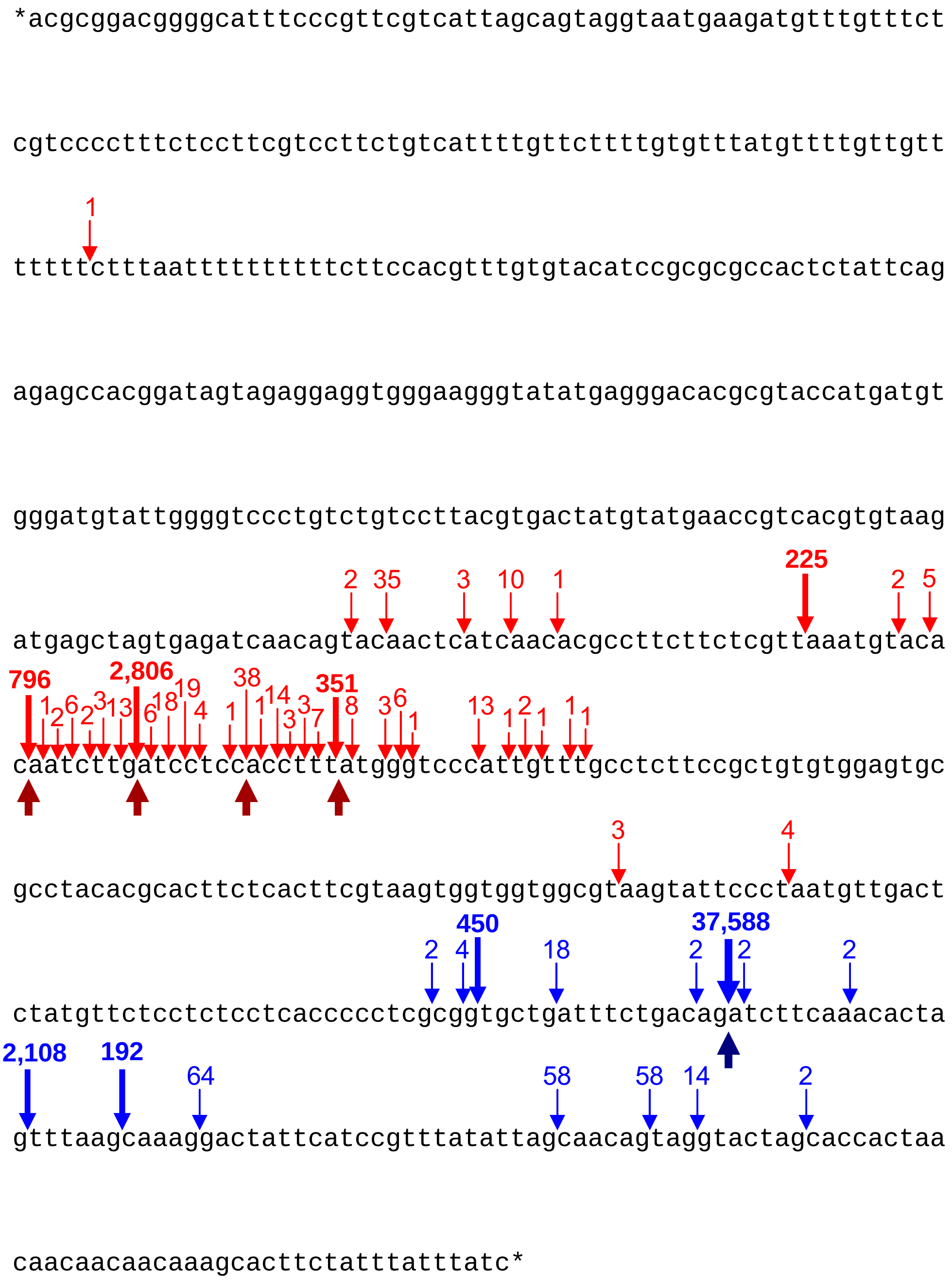

Figure S10. Comparison between RNA-seq mapped processing sites with sites mapped by previous sequencing of cDNA clones [8]. Polyadenylation sites and 3′-*trans*-splice sites mapped by cDNA clones sequencing in the β-tubulin/α-tubulin inter-ORF region are indicated by dark red and dark blue upward arrows, respectively. Polyadenylation sites and 3′-*trans*-splice sites mapped by end-reads in our RNA-seq for all β-tubulin/α-tubulin regions are indicated by red and blue downward arrows, respectively. The numbers on top of the arrows designate the number of reads. Asterisk at the beginning of the sequence indicates the β-tubulin stop codon and asterisk at the end of the sequence indicates the α-tubulin start codon.
