# Supplementary material for: The Transcriptome of the Human Pathogen Trypanosoma brucei at Single-Nucleotide Resolution
Source: PLoS Pathog. 2010 Sep 9;6(9):e1001090. doi: 10.1371/journal.ppat.1001090 (PMC2936537; doi:10.1371/journal.ppat.1001090)
Supplement: Figure S11 — Experimental validation of multiple primary trans-splice sites for T. brucei genes. Overlay of the number of reads (log2) from 5′-end- (blue) and 3′-end-enriched (red) libraries aligning to the shown regions of chromosomes IV (A–C) and V (D) covering the ORFs for Tb927.4.1020 (A), Tb927.4.1180 (B), Tb927.4.1600 (C) and Tb927.5.990 (D). SL-containing end-reads are shown as blue or red horizontal lines depending on their orientation (minus or plus strand, respectively). Dashed lines indicate the positions of a nested gene-specific primer and the expected positions of the 3′ trans-splice sites. Green bars indicate the potential products from an RT-PCR assay with SL and gene-specific primers with the predicted size of the fragments indicated on the left. (E) RT-PCR assay. Poly(A)+ RNA was reverse transcribed with random primers and the resulting cDNA was used as a template for nested PCR with an identical SL forward primer for both amplification steps. Tb927.5.990 is an example of a gene with highly homogeneous site for SL addition. (0.14 MB PDF) [file ppat.1001090.s011.pdf]

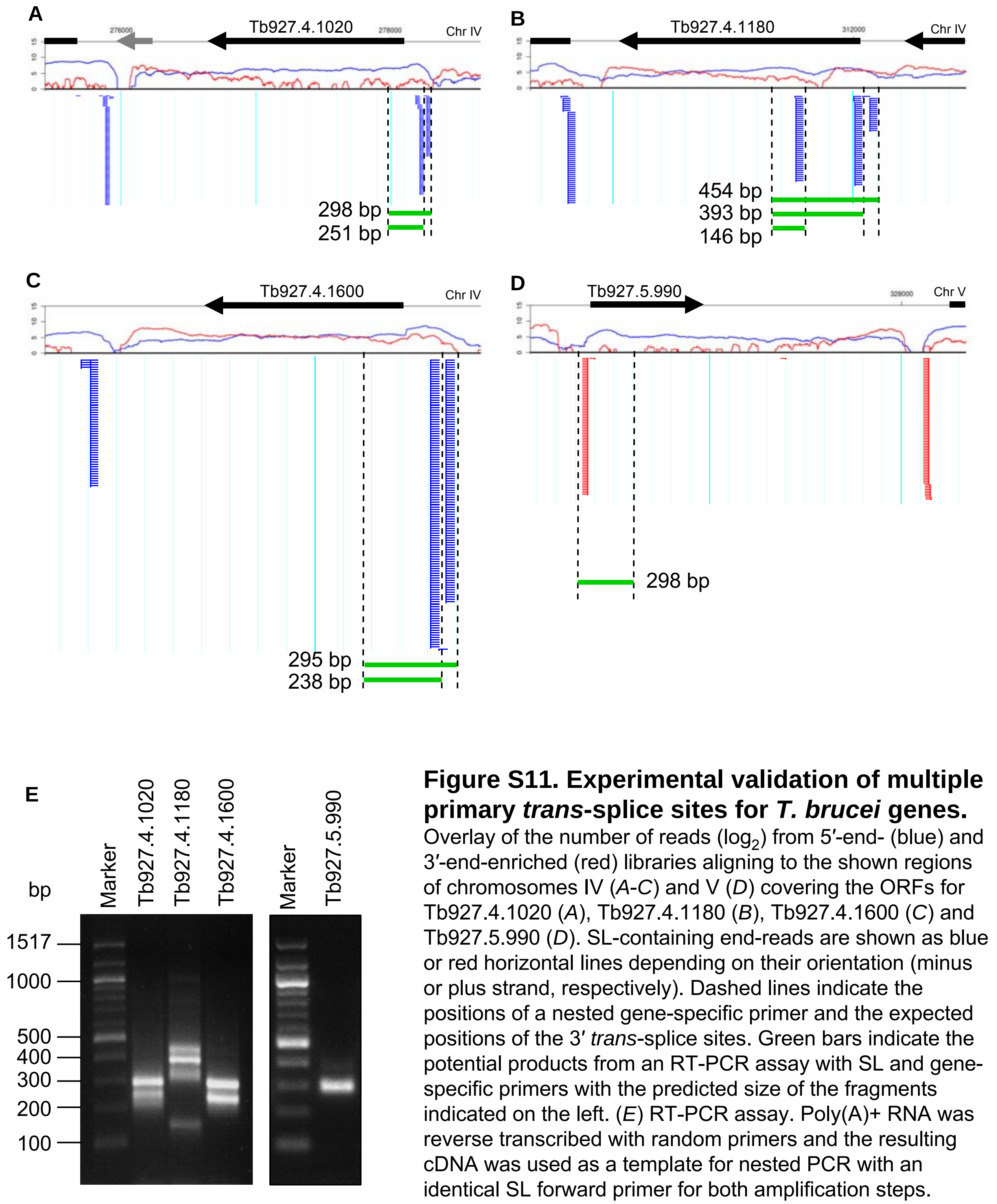


**Figure S11. Experimental validation of multiple primary *trans*-splice sites for *T. brucei* genes.** Overlay of the number of reads ($log_2$) from 5′-end- (blue) and 3′-end-enriched (red) libraries aligning to the shown regions of chromosomes IV (*A-C*) and V (*D*) covering the ORFs for Tb927.4.1020 (*A*), Tb927.4.1180 (*B*), Tb927.4.1600 (*C*) and Tb927.5.990 (*D*). SL-containing end-reads are shown as blue or red horizontal lines depending on their orientation (minus or plus strand, respectively). Dashed lines indicate the positions of a nested gene-specific primer and the expected positions of the 3′ *trans*-splice sites. Green bars indicate the potential products from an RT-PCR assay with SL and gene-specific primers with the predicted size of the fragments indicated on the left. (*E*) RT-PCR assay. Poly(A)+ RNA was reverse transcribed with random primers and the resulting cDNA was used as a template for nested PCR with an identical SL forward primer for both amplification steps. Tb927.5.990 is an example of a gene with highly homogeneous site for SL addition.
