# Supplementary material for: The Transcriptome of the Human Pathogen Trypanosoma brucei at Single-Nucleotide Resolution
Source: PLoS Pathog. 2010 Sep 9;6(9):e1001090. doi: 10.1371/journal.ppat.1001090 (PMC2936537; doi:10.1371/journal.ppat.1001090)
Supplement: Figure S12 — Outline of the protocol for generation of 5′-triphosphate-end-enriched library for RNA-Seq. Generation and sequencing of a cDNA library enriched for 5′-triphosphate RNA ends, the hallmark of a 5′ end generated by an RNA polymerase. (0.05 MB PDF) [file ppat.1001090.s012.pdf]

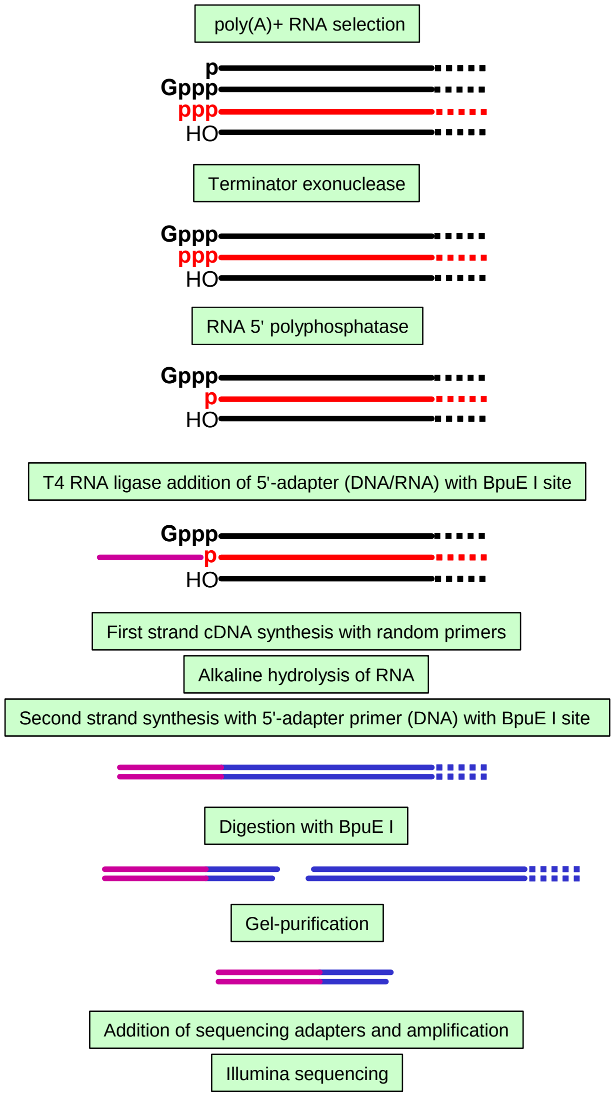

Figure S12. Outline of the protocol for generation of 5′-triphosphate-end-enriched library for RNA-seq.
