# Supplementary material for: The Transcriptome of the Human Pathogen Trypanosoma brucei at Single-Nucleotide Resolution
Source: PLoS Pathog. 2010 Sep 9;6(9):e1001090. doi: 10.1371/journal.ppat.1001090 (PMC2936537; doi:10.1371/journal.ppat.1001090)
Supplement: Figure S13 — Comparison between normalized (A) and non-normalized 5′-triphosphate-end-enriched library (B). Shown is a segment of chromosome VII surrounding a strand-switch region (SSR) of divergent transcription. Individual ORFs are indicated by bars colored based on their orientation. Grey arrows indicate the direction of transcription. The fold enrichment of reads (A) [(number of reads in the 5′-triphosphate-end-enriched library)×24/(number of reads in the 5′-end enriched library)] is plotted for the plus strand (red) and the minus strand (blue). The non-normalized number of reads (B) is shown for the plus strand (red) and the minus strand (blue). (0.17 MB PDF) [file ppat.1001090.s013.pdf]

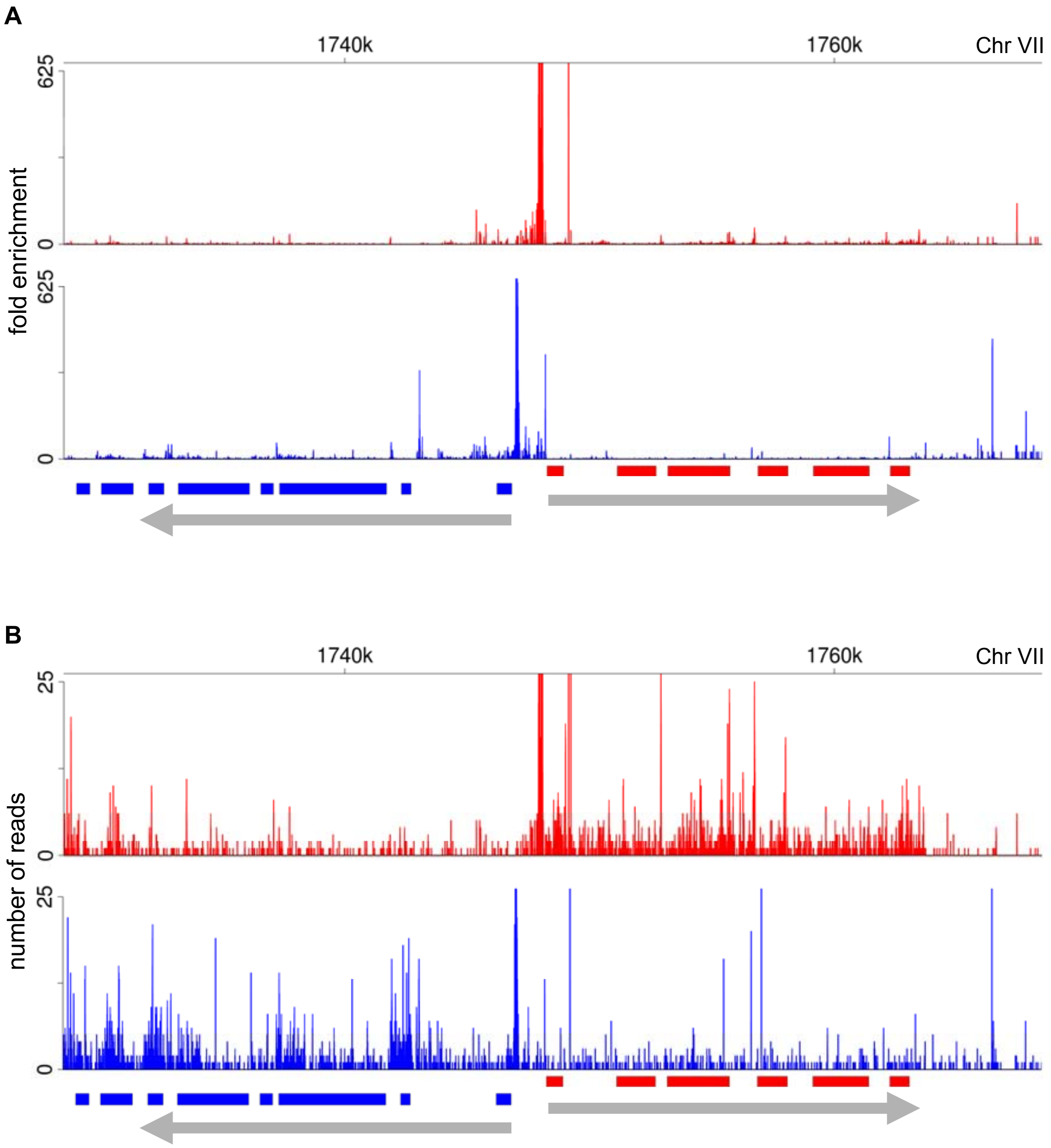

Figure S13. Comparison between normalized (*A*) and non-normalized 5′-triphosphate-end-enriched library (*B*). Shown is a segment of chromosome VII surrounding a strand-switch region (SSR) of divergent transcription. Individual ORFs are indicated by bars colored based on their orientation. Grey arrows indicate the direction of transcription. The fold enrichment of reads (*A*) [(number of reads in the 5′-triphosphate-end-enriched library) × 24 / (number of reads in the 5′-end enriched library)] is plotted for the plus strand (red) and the minus strand (blue). The non-normalized number of reads (*B*) is shown for the plus strand (red) and the minus strand (blue).
