# Supplementary material for: The Transcriptome of the Human Pathogen Trypanosoma brucei at Single-Nucleotide Resolution
Source: PLoS Pathog. 2010 Sep 9;6(9):e1001090. doi: 10.1371/journal.ppat.1001090 (PMC2936537; doi:10.1371/journal.ppat.1001090)
Supplement: Figure S14 — Comparison between the mapped splice sites and polyadenylation sites in this study and the data set in [17]. For the splice sites (A) and poly(A) sites (B), we defined a per-gene measure of overlap between the data in this study and the data in Siegel et al. 2010, as follows: define the entire set of sites found by both studies as si, for i = 1,…n. Define (in each study) the probability to observe the i'th site, pi, as the number of reads for that site divided by the total number of reads for the gene. The overlap is the sum over all sites of min(pi(this study), pi(Siegel et al 2010)). This gives 1, if there is perfect overlap and 0, if there is no overlap at all. (0.06 MB PDF) [file ppat.1001090.s014.pdf]

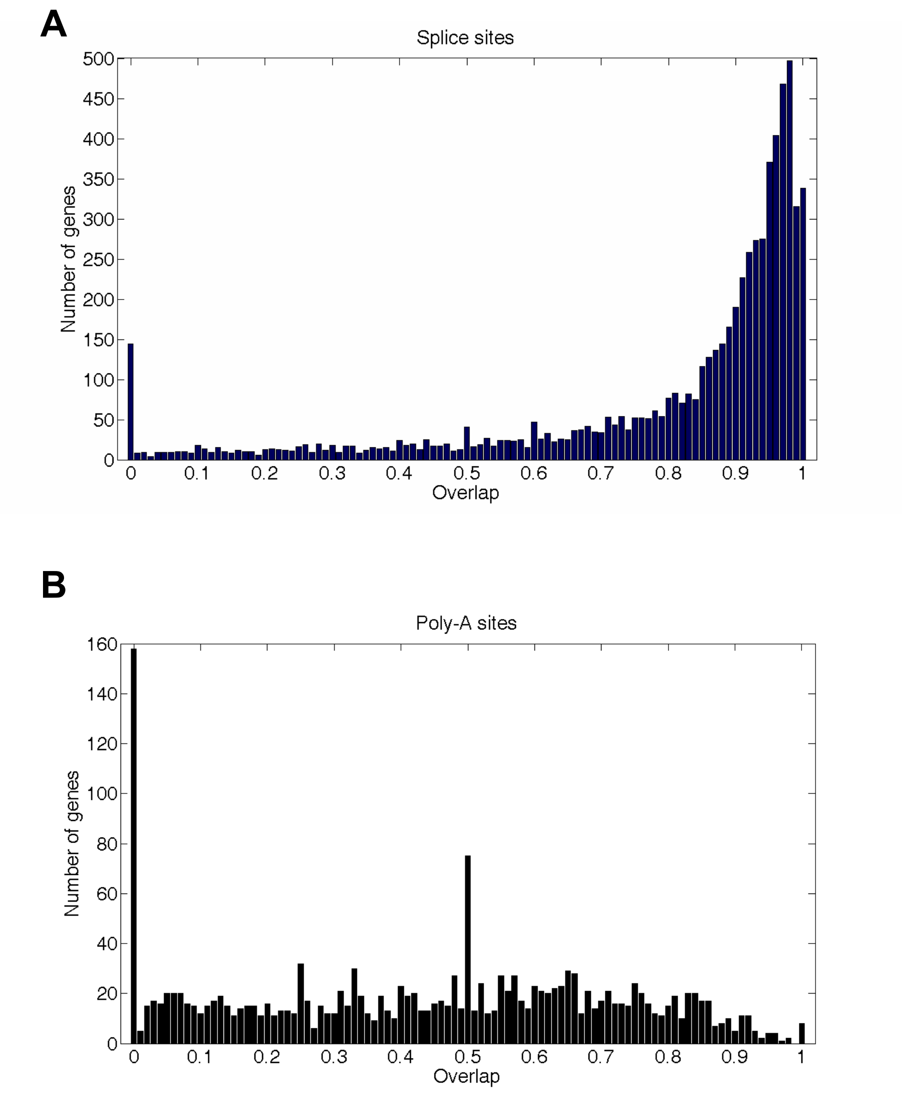

Figure S14. Comparison between the mapped splice sites and polyadenylation sites in this study and the data set in [17]. - For the splice sites (*A*) and poly(A) sites (*B*), we defined a per-gene measure of overlap between the data in this study and the data in Siegel et al. 2010, as follows: define the entire set of sites found by both studies as s_i, for i=1,...n. Define (in each study) the probability to observe the i'th site, p_i, as the number of reads for that site divided by the total number of reads for the gene. The overlap is the sum over all sites of min(p_i(this study), p_i(Siegel et al. 2010)). This gives 1, if there is perfect overlap and 0, if there is no overlap at all.
