# Supplementary material for: The Transcriptome of the Human Pathogen Trypanosoma brucei at Single-Nucleotide Resolution
Source: PLoS Pathog. 2010 Sep 9;6(9):e1001090. doi: 10.1371/journal.ppat.1001090 (PMC2936537; doi:10.1371/journal.ppat.1001090)
Supplement: Figure S15 — Abundance comparison between the data set in this study and that in [17]. Pairwise (gene-by-gene) comparison of RNA-Seq-based gene abundance reported previously with those derived from the current study (tabulated in Table S6). Correlation coefficients between the sets are 0.697 (Pearson) and 0.483 (Spearman). (0.05 MB PDF) [file ppat.1001090.s015.pdf]

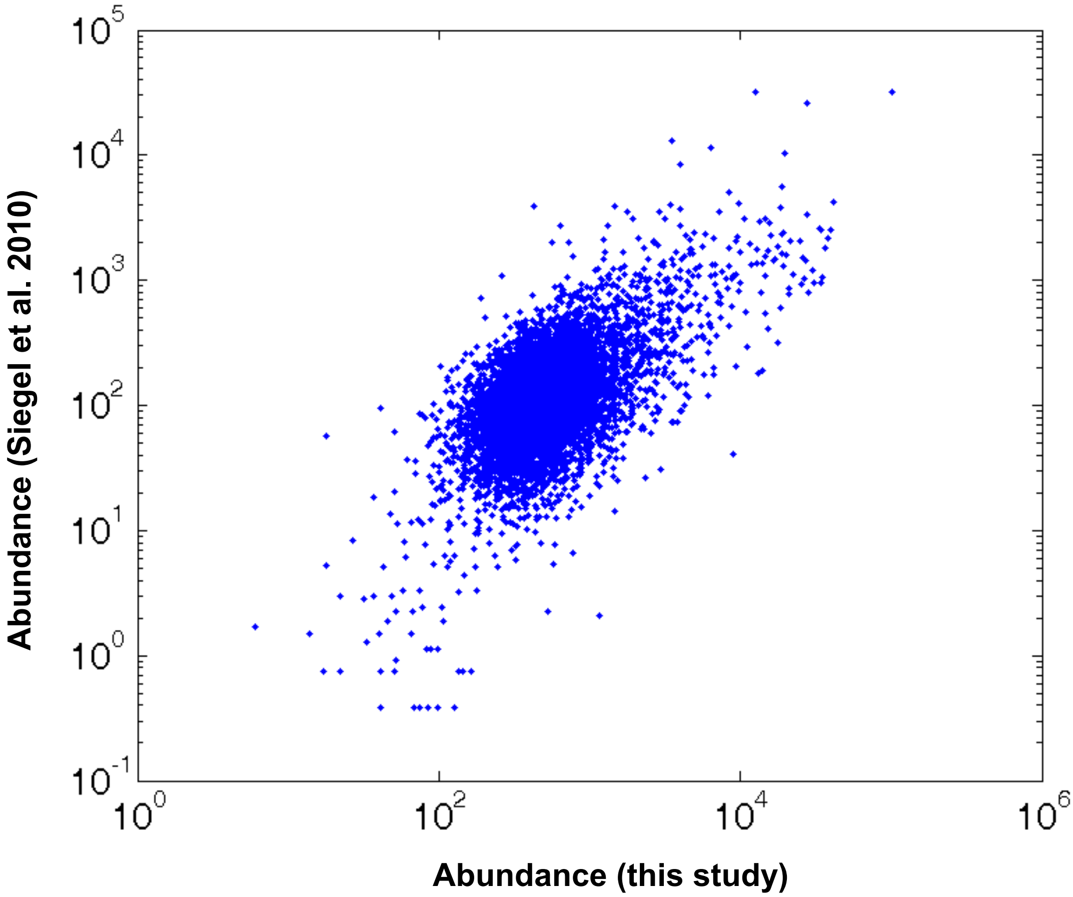

Figure S15. Abundance comparison between the data set in this study and that in [17]. - Pairwise (gene-by-gene) comparison of RNA-Seq-based gene abundance reported previously with those derived from the current study (tabulated in Table S6). Correlation coefficients between the sets are 0.697 (Pearson) and 0.483 (Spearman).
