# Supplementary material for: The Transcriptome of the Human Pathogen Trypanosoma brucei at Single-Nucleotide Resolution
Source: PLoS Pathog. 2010 Sep 9;6(9):e1001090. doi: 10.1371/journal.ppat.1001090 (PMC2936537; doi:10.1371/journal.ppat.1001090)
Supplement: Supporting Information S1 — Additional description of materials and methods. (0.17 MB PDF) [file ppat.1001090.s016.pdf]

Additional detailed Methods

Preparation of cDNA libraries for Illumina sequencing

3′-end-enriched libraries

Poly(A)$^{\pm}$ RNA selection

Total RNA was prepared with Trizol reagent (Invitrogen) according to manufacturer's instructions. Any genomic DNA contamination was removed by treatment of 100 μg of the total RNA with 1 U RQ1 RNase-free DNase I (Promega) for 15 min at 37°C in 100 μL reaction containing 40 mM Tris-HCl, pH 8.0, 10 mM $MgSO_4$ and 1 mM $CaCl_2$. The reaction was extracted with equal volumes of buffer-saturated phenol (pH 7.5) and chloroform. The RNA was subjected to two rounds of poly(A)$^+$ selection with Oligotex mRNA mini kit (Qiagen) according to the manufacturer's instructions with the following modifications: binding of RNA to the resin was performed on ice and all centrifugation steps were performed at 15°C. Elution after the first round of selection was with 2× 100 μL elution buffer and, after the second round, with 25 μL elution buffer.

First strand cDNA synthesis

The eluted poly(A)$^+$ RNA (~500 ng in 10.5 μL) was mixed with 1 μL (500 ng) random hexadeoxynucleotide primers (Promega) or the same amount of 5′-T_{15}VN-3′ oligonucleotide (IDT, custom DNA oligo; V=A, G or C; N=T, A, G or C), incubated for 5 min at 65°C and chilled on ice. To each reaction were added 7.5 μL of mixture containing 4 μL 5× first-strand buffer (250 mM Tris-HCl, pH 8.3, 375 mM KCl, 15 mM $MgCl_2$)(Invitrogen), 2 μL 0.1 M DTT, 1 μL 10 mM dNTPs and 0.5 μL (20 U) Protector RNase inhibitor (Roche). The random hexamers reaction was incubated for 2 min at 25°C and the T_{15}VN reaction for 2 min at 42°C. Two hundred units (1 μL) SuperScript II reverse transcriptase (Invitrogen) were added, the random hexamers reaction was incubated for 10 min at 25°C, and both reactions were incubated for 50 min at 42°C. The enzyme was inactivated with 15 min incubation at 70°C.

Second strand cDNA synthesis

To the first strand synthesis reactions (after chilling on ice and brief centrifugation) were added 61 μL water, 10 μL 10× NEBuffer 2 (100 mM Tris-HCl, pH 7.9, 500 mM NaCl, 100 mM $MgCl_2$, 10 mM DTT)(New England Biolabs), 3 μL 10 mM dNTPs, 0.5 μL (2.5 U) RNase H (New England Biolabs) and 5 μL (50 U) *E. coli* DNA polymerase I (New England Biolabs). After incubation for 2 hours at 16°C, 3 μL (9 U) of T4 DNA polymerase (New England Biolabs) were added and the reactions was incubated for 2 min at 25°C. The synthesized cDNA was purified on NucleoSpin Extract II columns (Macherey-Nagel) and eluted in 45 μL of the provided elution buffer.

cDNA fragmentation

Three separate reactions were assembled for each of the cDNA samples. Each contained 15 μL of the purified cDNA in a total volume of 20 μL containing 20 mM Tris-HCl, pH 7.5, 1 mM $CaCl_2$, 10 mM $MnCl_2$ and 1×10^{-2}, 2×10^{-3} or 1×10^{-3} U of RQ1 DNase I

(Promega). The reactions were incubated for 10 min at room temperature, followed by immediate extraction with a mixture of phenol:chloroform:isoamyl alcohol (25:24:1). The fragmented cDNA from the 3 reactions was combined for each sample.

<u>Size-selection of cDNA fragments</u>
Approximately half of the fragmented cDNA (30 μL) was run on 1% agarose gel in 0.5×TBE alongside 100 bp DNA ladder (Invitrogen). After the marker bands separated well (xylene cyanol dye has migrated ~1.8 cm inside the gel), ~2 mm thick gel slice from each sample lane was cut out very carefully, containing cDNA fragments corresponding in size to the 200 bp marker band (the cDNA is not visible with ethidium bromide staining). Care was taken not to cross-contaminate samples. The DNA was purified from the gel slice on NucleoSpin Extract II column (Macherey-Nagel) and eluted in 35 μL of the provided elution buffer.

<u>End-repair</u>
The ends of the selected cDNA fragments were repaired in a 50 μL reaction with the End-It DNA end-repair kit (Epicentre) according to the manufacturer's instructions. The DNA was purified on NucleoSpin Extract II column (Macherey-Nagel) and eluted in 33 μL of the provided elution buffer.

<u>Addition of a single dA at the 3′ ends</u>
In a 50 μL reactions were combined the eluted cDNA, 5 μL 10× NEBuffer 2 (100 mM Tris-HCl, pH 7.9, 500 mM NaCl, 100 mM $MgCl_2$, 10 mM DTT)(New England Biolabs), 10 μL 1 mM dATP and 3 μL (15 U) Klenow fragment (3′-5′ exo^-)(New England Biolabs). The reactions were incubated for 30 min at 37°C. The DNA was purified on NucleoSpin Extract II columns (Macherey-Nagel) and eluted in 23 μL of the provided elution buffer.

<u>Preparation of forked adapters</u>
The adapters were annealed in a 50 μL reaction, by mixing 10 μL of 50 mM Tris-HCl, pH 7.5, 50 mM NaCl, 20 μL 100 μM first genomic adapter (Illumina, Inc. All rights reserved.) and 20 μL 100 μM second genomic adapter (Illumina, Inc. All rights reserved.). The mixture was heated for 1 min at 97°C and very slowly cooled to room temperature. The tube was briefly centrifuged and this 40 μM adapters stock was stored at -20°C.

<u>Ligation of adapters</u>
The annealed adapters were diluted to 10 μM with 10 mM Tris-HCl, pH 7.5, 10 mM NaCl. A 50 μL reaction was assembled by combining the eluted DNA from the last step (single A addition), 25 μL 2× Ligation buffer (Promega, LigaFast rapid DNA ligation system), 1 μL 10 μM adapters and 2 μL (6 U) T4 DNA ligase (Promega, LigaFast rapid DNA ligation system). Ligation proceeded for 15 min at room temperature. The ligated DNA was purified on NucleoSpin Extract II columns (Macherey-Nagel) and eluted in 41 μL of the provided elution buffer.

PCR amplification of the cDNA libraries

The libraries were enriched by PCR in 50 μL reactions containing 10 μL (~1/4th of the eluted ligated DNA, 5 μL 10× Pfx amplification buffer (Invitrogen), 2 μL 25 μM first genomic PCR Primer (Illumina, Inc. All rights reserved.), 2 μL 25 μM second genomic PCR Primer (Illumina, Inc. All rights reserved.), 2 μL 50 mM $MgSO_4$, 2 μL 10 mM dNTPs and 0.8 μL (2 U) platinum Pfx DNA polymerase (Invitrogen).

Program:

1. 5 min at 94°C
2. 15 sec at 94°C
3. 30 sec at 65°C
4. 30 sec at 68°C
5. go 15 times to step 2.
6. 5 min at 68°C
7. hold at 4°C.

Analyse 5-10 μL by 1% agarose gel electrophoresis.

cDNA library purification

The amplified cDNA product was purified on 1% agarose gel, purified from the gel on NucleoSpin Extract II column (Macherey-Nagel), eluted in 100 μL and subjected to a second round of purification on NucleoSpin Extract II column. The final sample was eluted in 30 μL of the provided elution buffer. The approximate DNA concentration was estimated by running a small amount of the sample on agarose gel alongside markers of known concentration, and the precise concentration was determined by an Agilent Bioanalyzer.

5′-end-enriched libraries

mRNA enrichment

Total RNA was prepared with Trizol reagent (Invitrogen) according to manufacturer's instructions. 10 μg (or less) of the total RNA were treated with 1U Terminator 5′-phosphate-dependent exonuclease (EPICENTRE) in a 20 μL reaction for 1 hr at 30°C according to manufacturer's instructions. After the 1hr incubation, 1 U RQ1 RNase-free DNase I (Promega) was added and the mixture was incubated for 5 min at 37°C. The reaction was diluted to 100 μL with water and extracted with equal volumes of buffer-saturated phenol (pH 7.5) and chloroform. The RNA was precipitated with 10 μL 2.5 M sodium acetate (pH 5.0), 2 μL GlycoBlue (Ambion) and 300 μL ethanol for more than 3 hrs at -20°C. After centrifugation for 15-20 min at 13,200 rpm in a refrigerated rotor, the pellet was washed with 700 μL 70% EtOH, air-dried and dissolved in 10.5 μL water.

First strand cDNA synthesis

Performed with random primers (see above).

Second strand cDNA synthesis

To the first strand synthesis reaction were added 7 μL 1 N NaOH and the reaction was incubated for 15 min at 65°C. Six μL 1 M Tris-HCl (pH 8.0), 6.5 μL 1 N HCl and 4 μL 3 M sodium acetate were added with mixing after each addition. The cDNA was

precipitated with 1 μL GlycoBlue (Ambion) and 120 μL ethanol at -20°C, the precipitate was washed with 70% EtOH and dissolved in 66 μL water. To the DNA solution were added 10 μL 10× Pfx amplification buffer (Invitrogen), 10 μL PCR enhancer solution (Invitrogen), 5 μL 20 pmol/μL SL Primer 5′-GCTATTATTAGAACAGTTTCTGTACTATATTG -3′ (IDT, custom DNA oligo), 4 μL 50 mM $MgSO_4$, 4 μL 10 mM dNTPs and 1 μL (2.5 U) platinum Pfx DNA polymerase (Invitrogen). The reaction was incubated in a thermocycler: step 1- 7 min at 94°C; step 2- 5 min at 40°C; step 3- 20 min at 68°C; step 4- hold at 4°C. The cDNA was purified on NucleoSpin Extract II column (Macherey-Nagel) and eluted in 45 μL of the provided elution buffer.
cDNA fragmentation and the rest of the protocol were performed as described above.

5′-triphosphate end-enriched libraries

Total RNA (500 μg) was subjected to two rounds of poly(A)$^+$ selection as described above and eluted in 35 μL elution buffer. The RNA was treated with 2 U Terminator 5′-phosphate-dependent exonuclease (EPICENTRE) in a 40 μL reaction for 1 hr at 30°C according to manufacturer's instructions. The reaction was diluted to 100 μL with water and extracted with equal volumes of buffer-saturated phenol (pH 7.5) and chloroform. The RNA was precipitated with 10 μL 2.5 M sodium acetate (pH 5.0), 1 μL GlycoBlue (Ambion) and 300 μL ethanol for more than 3 hrs at -20°C. After centrifugation for 15-20 min at 13,200 rpm in a refrigerated rotor, the pellet was washed with 750 μL 70% EtOH, air-dried and dissolved in 34 μL water. The RNA was treated with 40 U RNA 5′-polyphosphatase (EPICENTRE) in a 40 μL reaction for 30 min at 37°C according to manufacturer's instructions. The RNA was extracted with phenol, precipitated with ethanol, washed and dried as described above and dissolved in 15 μL water. A 5′-adapter with BpuE I site, 5′-GCACCATATAACCGCTTCCrUrUrGrArG-3′ (IDT, custom DNA oligo) was ligated overnight to the available 5′-monophosphate ends in a 20 μL reaction at 4°C. The reaction contained 200 pmol 5′-adapter, 1× RNA ligase buffer (Ambion) and 10 U T4 RNA ligase (Ambion). The RNA was extracted with phenol, precipitated with ethanol, washed, dried and used as a template for first strand cDNA synthesis as described above. To the first strand synthesis reaction were added 7 μL 1 N NaOH and the reaction was incubated for 15 min at 65°C. Six μL 1 M Tris-HCl (pH 8.0), 6.5 μL 1 N HCl and 4 μL 3 M sodium acetate were added with mixing after each addition. The cDNA was precipitated with 1 μL GlycoBlue (Ambion) and 120 μL ethanol at -20°C, the precipitate was washed with 70% EtOH and dissolved in 66 μL water. To the DNA solution were added 10 μL 10× Pfx amplification buffer (Invitrogen), 10 μL PCR enhancer solution (Invitrogen), 5 μL 20 pmol/μL BpuE I Primer 5′-GCACCATATAACCGCTTCCTTGAG-3′ (IDT, custom DNA oligo), 4 μL 50 mM $MgSO_4$, 4 μL 10 mM dNTPs and 1 μL (2.5 U) platinum Pfx DNA polymerase (Invitrogen). The reaction was incubated in a thermocycler: step 1- 7 min at 94°C; step 2- 5 min at 46°C; step 3- 20 min at 68°C; step 4- hold at 4°C. The cDNA was purified on NucleoSpin Extract II column (Macherey-Nagel) and all DNA fragments larger than 100 bp were purified on 1.2% agarose gel which was run for a short time, but enough to separate a 100 bp marker band from the rest of marker fragments. The purified DNA was

digested with 25 U BpuE I (New England BioLabs) in the presence of *S*-adenosylmethionine according to manufacturer's instructions in a 100 μL reaction for 2 hrs at 37°C. The DNA was purified on NucleoSpin Extract II column (Macherey-Nagel), loading the column twice with the same sample, and separated on 2% agarose gel alongside 100 bp ladder and pBR322 DNA-Msp I digest marker (New England BioLabs). Several thin gel segments covering the ~40 bp size bands along the length of the gel were excised and the DNA from each slice was purified on NucleoSpin Extract II column (Macherey-Nagel). End repair, a single dA, Illumina adapter ligation and PCR amplification was performed separately on all samples as described above. Sequencing was performed on the amplified product of the right expected size (130 bp) resulting from one of the samples.

Northern blotting

Total RNA or RNA after one round of oligo(dT) selection was fractionated on 1.2% agarose gels in the presence of 6.3% formaldehyde in 40 mM 3-morpholinopropane-1-sulfonic acid (MOPS) and 2 mM EDTA. RNA ladder 0.5-10 kb (Invitrogen) was used as size markers. The RNA was transferred to a Hybond-N nylon membrane (GE Healthcare) by capillary transfer with 10× SSC, UV cross-linked to the membrane and stained with methylene blue. After pre-hybridization for 1 hr in 5× SET pH 7.4, 10× Denhardt's, 1% SDS and 100 μg mL^{-1} yeast RNA, the membrane was hybridized overnight in the same solution to DNA probes that were either internally labeled by synthesis with specific dsDNA template, antisense primer and Pfu DNA polymerase (Invitrogen) in the presence of [α-^{32}P]dCTP, or end-labeled synthetic ~85 nt antisense oligodeoxyribonucleotides with T4 polynucleotide kinase (New England BioLabs) and [γ-^{32}P]ATP. The membrane was washed 2-3 times (30 min each) with 2× SSC, 0.1% SDS and hybridization signal was detected by PhosphorImager.

Enzyme assay for RNA 5′-end analysis

Total RNA was prepared with Trizol reagent (Invitrogen) according to manufacturer's instructions. Any genomic DNA contamination was removed by treatment of 100 μg of the total RNA with 1 U RQ1 RNase-free DNase I (Promega) for 15 min at 37°C in 100 μL reaction containing 40 mM Tris-HCl, pH 8.0, 10 mM $MgSO_4$ and 1 mM $CaCl_2$. The reaction was exracted with equal volumes of buffer-saturated phenol (pH 7.5) and chloroform. The RNA was precipitated with 10 μL 2.5 M sodium acetate (pH 5.0) and 300 μL ethanol for more than 3 hrs at -20°C. After centrifugation for 15-20 min at 13,200 rpm in a refrigerated rotor, the pellet was washed with 1 mL 70% EtOH, air-dried and dissolved in 100 μL water. Reactions with 5′-end-modifying enzymes were performed in a volume of 40 μL with 14 μg total RNA with 40 U RNA 5′-polyphosphatase (EPICENTRE), 40 U tobacco acid pyrophosphatase (EPICENTRE), 10 U T4 polynucleotide kinase (New England BioLabs) plus 1 mM ATP, or 1 U alkaline phosphatase (Roche) according to manufacturer's instructions . Control reaction contained only RNA and 1× buffer for RNA 5′-polyphosphatase (EPICENTRE). After the appropriate incubation, reactions were diluted to 100 μL with water and extracted with equal volumes of buffer-saturated phenol (pH 7.5) and chloroform. The RNA was precipitated with 10 μL 2.5 M sodium acetate (pH 5.0), 1 μL GlycoBlue (Ambion) and 300 μL ethanol for more than 3 hrs at -20°C. After centrifugation and wash with 750 μL

75% EtOH, each RNA pellet was air-dried, dissolved in 35 μL water, split evenly between two tubes. In one of the tubes the RNA was treated with 1 U Terminator 5′-phosphate-dependent exonuclease (EPICENTRE) in a 20 μL reaction for 1 hr at 30°C according to manufacturer's instructions. The second tube served as a control and was incubated under the same conditions only in the presence of 1× buffer for Terminator (EPICENTRE). RNA was extracted with phenol and chloroform and precipitated with ethanol. Reverse transcription with random hexamers was performed as described above and the resulting cDNA was used as a template for PCR with forward and reverse primers specific for the transcripts of interest.

RT-PCR

Poly(A)$^+$ RNA was used as a template for cDNA synthesis as described above. The first PCR was performed with forward SL primer 5′-GCTATTATTAGAACAGTTTCTGTACTATATTG -3′ (IDT, custom DNA oligo) and a gene-specific reverse primer. The nested PCR was carried out with the same forward SL primer, with a nested gene-specific primer and 0.2 μL of the first 50 μL PCR as template. Products were separated on native 1% agarose gels alongside 100 bp DNA ladder (New England Biolabs).

Identification and analysis mRNA ends from 5′- and 3′-end-enriched libraries

<u>Processing of 5′ end-reads</u>

We identified reads putatively overlapping with the ends of sequenced transcripts. Over 2.5 million reads from the SL-primed library--or about 7.7% of all reads from this set--contained the entire splice-leader sequence at their 5′ ends. This 32-nucleotide sequence (GCTATTATTAGAACAGTTTCTGTACTATATTG) was removed from the original 75 nt read, leaving 43 nucleotides remaining. The first 28 nucleotides of the newly trimmed reads were aligned to the genome reference, with a maximum of 2 allowed mismatches and one alignment reported per read. Over 94% (~ 2.4×10^6) of the SL-containing reads aligned successfully to the 11 major chromosomes.

<u>Processing of 3′ end-reads</u>

A more complex method was used for the determination of polyadenylation sites, because the contiguous A nucleotides that signal an end-nucleotide offer less confidence than the highly unique SL sequence. First, reads consisting primarily of A or T nucleotides (more than 31 out of 35 total nucleotides), a frequent artifact of Illumina sequencing, were removed from the oligo(dT) libraries. Next, contiguous A or T stretches, 5-15 letters long, were trimmed off the 5′ or 3′ ends of the sequences, respectively. Trimmed reads, ranging in length from 15-30 nucleotides, represented putative 3′-end reads and were aligned to the genome reference. In order to distinguish poly-A tails from RNA transcribed from contiguous As in the genome, alignments were only considered to represent poly-adenylation sites if the putative end-read contained a longer stretch of A's than was present in the genome at the alignment locus.

Analysis of 5′-triphosphate end-enriched libraries

Preparation of the 5′-triphosphate end-enriched library produced two sets of reads, categorized by the position of the 14-nt sequence representing the 5′ transcript end (the "end sequence"). The first set, corresponding to the top strand portrayed in the final step of Figure S12, contained the 24 nt adapter (GCACCATATAACCGCTTCCTTGAG) at the beginning of the read, followed by the end sequence, spanning nucleotides 25 through 38. The second set, corresponding to the bottom strand in Figure S12, contained the reverse complement end sequence in the left-most 14 nt, followed by the reverse complement of the same 24 nt adapter. These 14 nt end sequences were extracted from the two sets of reads, and the second set was reverse complemented, to produce the end tags. These end-tags were aligned to the genome with no mismatches allowed, and one alignment reported per tag. Out of 31.8×10^6 total reads, 33% contained extractable end-tags. Of these, 57% (6.1×10^6) aligned to the genome, 18% uniquely.

All read manipulations, as well as the genome-wide site analyses, were performed with custom scripts written either in Perl or for a combination of the R statistical software with the bioinformatics-centric Bioconductor tools installed.

Measurement of transcript abundance

A relative measure of transcript abundance was derived from the number of reads (not including end-reads) that align within a 500-nucleotide window, extending into the gene from the 5′ end of the transcript for the SL-library and from the 3′ end for the poly(A) library.

Polypyrimidine tract calculations

The polypyrimidine tract was identified as the longest stretch of pyrimidines separated by no more than one purine in a 200 nt window upstream of the splice site with the maximal number of reads. If two tracts had the same length, the one nearest to the splice site was chosen.

Novel ORF analysis

Novel transcripts identified in our analysis were searched for ORFs to determine their polypeptide coding potential. All non-overlapping ORFs were extracted for further analysis, including assessing them for the presence of predicted signal peptides (using SignalP - www.cbs.dtu.dk/services/SignalP; [54]).

Intron analysis

We used TopHat [23] to identify putative *cis*-splice junctions, based on the presence of junction-spanning reads in the 5′-end-enriched library only, and not on coverage gaps (a deviation from the TopHat default). All putative introns between 30 and 5,000 bp were then considered by manual inspection.
